# Identification of plant-based spilled oils using direct analysis in real-time–time-of-flight mass spectrometry with hydrophobic paper sampling

**DOI:** 10.1007/s10661-024-13583-1

**Published:** 2025-01-14

**Authors:** Paige McCallum, Genesis Saturos, Lola Rabinovitch, Taylor Filewood, Honoria Kwok, Jeffrey Yan, Robert Cody, Pamela Brunswick, Dayue Shang

**Affiliations:** 1 Science and Technology Branch, Pacific Environmental Science Centre, Environment and Climate Change Canada, Pacific and Yukon Laboratory for Environmental Testing, North Vancouver, BC Canada; 2 JEOL USA Inc, Peabody, MA USA

**Keywords:** Direct analysis in real-time time-of-flight mass spectrometry (DART-TOFMS), Oil spill forensics hydrophobic paper sampling, Principal component analysis (PCA), Discriminant analysis of principal components (DAPC), Plant-based oil spill

## Abstract

Spilled plant-based oils behave very differently in comparison to petroleum oils and require different clean-up measures. They do not evaporate, disperse, dissolve, or emulsify to a significant degree but can polymerize and form an impermeable cap on sediment, smothering benthic media and resulting in an immediate impact on the wildlife community. The current study explored the application of rapid up-to-date direct analysis in real time (DART) with high-resolution mass spectrometry for plant-based oil typing. The study introduced a new concept of using hydrophobic paper to collect and analyze oil samples, thus minimizing sample preparation and expenses. Application of this technique showed its ability to speedily distinguish plant-based from petroleum-based oils. A microcosm experiment exposing plant-based oil samples to weathering processes for comparison with petroleum-based oils demonstrated the ability of the method to classify weathered oil samples and identify their source oil. It was observed that canola and peanut oil were the most resistant to weathering processes. The developed DART-TOFMS method was shown to be accurate for short-term weathered oil spills up to between 12 and 26 days of exposure. The developed method performed identification in less than a day compared to the established multi-day method for oil spill forensics requiring careful sample collection in glass containers, time-consuming laboratory clean-up, lengthy gas chromatography sequences, and careful integration including integration of retention time markers.

**Supplementary Information:**

The online version contains supplementary material available at 10.1007/s10661-024-13583-1.

## Introduction

As the world population increases every year (United States Census Bureau World Population Estimated at 8, 2023), the demand for food resources and sustainable energy also increases (Fry & Fitton, 2010). The known contribution of fossil fuel combustion to global warming inevitably means that the search for alternative energy is on the rise (McCormick & Moriarty, 2023; Barrett et al*.,* 2015). Though plant-based oils have previously been considered only as food, there is a large transition to their use as biofuel in place of fossil fuels (Lu et al*.,* 2011; Fry & Fitton, 2010). The rising demand for plant-based oil and its many applications has led to a significant increase in production and consumption (Lu et al*.,* 2011; Fry & Fitton, 2010). As oil-based exports rise, the risk of spillage also increases, particularly in relation to the main bulk transport routes by marine vessels (Fingas, 2014; Tamothran, et al*.* 2022; Al-Darbin et al., 2005; Mudge, 1995). Several spills in the past five decades have demonstrated the deleterious effects plant-based oils have on marine environments (Fingas, 2014; Tamothran et al*.,* 2022; Al-Darbin, et al*.* 2005; Mudge, 1995). In 2000, approximately 200 t of canola oil spilled into Vancouver Harbor, British Columbia, resulting in over 200 waterfowl casualties (Fingas, 2015). Nutrient availability and biodiversity can also be impacted by the presence of plant-based oils; a total of 250 t of sunflower oil spilled in the Con Joubert Bird Sanctuary, South Africa, resulting in a shift in phytoplankton taxonomic composition, which is considered to be due to oil-caused turbidity (Oberholster et al., 2010).

When plant-based oil is spilled in a marine environment, as per petroleum-based biofuel oils, the oil is subjected to natural degradative processes known as weathering. Weathering processes, such as photooxidation, evaporation, biodegradation, photodegradation, and dissolution, all affect the chemical components of an oil. However, the processes and results involved differ for the plant-based oils versus petroleum-based biofuels, with the latter containing more highly toxic components. For example, the weathering processes of plant-based oils resulted in shoreline malodor (Non-Petroleum Oil Spills, 2019), and some oils were able to polymerize to form an impermeable cap on the sediment, subsequently smothering benthic media (Non-Petroleum Oil Spills, 2019; Al-Darbin, et al*.* 2005; Mudge, 1995; Mudge, 1997). Though not directly toxic, the immediate impact of such smothering causes a shift in the wildlife community and can cause long-term degradation, making plant-based oils a major hazard to the environment (Al-Darbin, et al*.* 2005; Mudge, 1995; Mudge, 1997; Salam et al*.,* 2012). In fact, the International Convention for the Prevention of Pollution from Ships (MARPOL) has classified many plant-based oils as Category Y noxious; plant-based oils are a hazard to marine resources, human health, and amenities when introduced to the sea (International Maritime Organization, 2024; Al-Darbin, et al*.* 2005; Mudge, 1995).

As a result of weathering, the identification of oil type and source, which is critical to guide the clean-up procedure, becomes complex. Given the negative impacts and the growing environmental risks of plant-based oil spills, a reliable and rapid method for identifying spills to their source is needed in order to assign responsibility and inform clean-up response. It is important to quickly identify if the spilled oil is plant-based or petroleum oil, as both oil types exhibit different behaviors in marine environments. The European Committee for Standardization (CEN) EN 15522–2 has established a method for characterizing and identifying petroleum oil spill samples using GC-FID and GC-low-resolution MS. The GC–MS method is regarded as the gold standard for petroleum oil identification through its use of biomarker ion ratio comparison between spill samples and source oils (Tikkisetty et al*.,* 2023a, b; Chua et al., 2020). While this method has only recently been expanded to fatty acid monitoring, well-established studies on plant oil forensics are scarce; most published literatures are focused on the detection of adulteration and quality control (Vaclavik et al., 2009; Shang et al*.,* 2018; Vaclavik et al., 2023). A previous publication discussed a case of successfully matching plant-based oil spills to their source using liquid chromatography-tandem mass spectrometry (Shang et al*.,* 2018). Spill samples were identified to the source by the presence of coloring agents, additives, and characteristic compounds. Sample preparation prior to analysis by GC–MS or LC–MS employed liquid–liquid extraction of samples using acetonitrile and hexane (Shang, et al*. *2018). While the reported study highlighted the advantages of LC–MS over GC-FID and GC–MS for analyzing hydrophilic organic compounds, the process was time-consuming and laborious, and the complexity of data processing demanded senior analyst experience (Tikkisetty et al*.,* 2023a, b; Brunswick et al., 2025).

As an alternative to the previously discussed analytical methods for plant oil identification, direct analysis in real-time–time-of-flight mass spectrometry (DART-TOFMS) holds significant potential. In fact, no sample preparation is needed prior to the analysis of the oil, allowing for a quicker turnaround time. In the current oil testing methodologies (CEN), the marine sample is collected in a glass container for transport to the testing facility. The use of fragile glass is cumbersome to the field sampling team and risks the integrity of the sample with frequent breakage of glass containers. Taking advantage of the unique feature of DART-TOFMS analysis, this study tackled the problem by alternatively using hydrophobic paper to collect the oil sample while repelling water. The paper with collected oil can be transported in a Ziploc™ bag, minimizing the space and resources of the sampling process.

Further to the simplicity of using hydrophobic paper for plant oil introduction, the application of DART-TOFMS additionally avoids the lengthy preparation required for GC–MS and LC–MS analyses. The DART atmospheric pressure ion source utilizes interactions between a sample and metastable gas atoms to ionize chemical compounds from a surface (Robert et al*.,* 2001). Ionization by DART does not require specialized sample inlet streams with enclosed ion sources, and its unique ionization method allows for the analysis of solids, liquids, and gases. Studies using the DART instrument have proven its ability to employ collected mass spectra to identify wood species, petroleum oils, drugs, and species-specific turtle oil (Price et al*.,* 2022; Tikkisetty et al*.,* 2023a, b; Shang et al., 2018; Espinoza et al*.,* 2021). The mass spectra, for example, from endangered wood species or various petroleum oil types, have been collated and used to determine an unknown sample’s “chemical fingerprint.” Heatmaps created from sample spectra show the relative abundance of ions in a mass spectrum, allowing analysts to visually compare sample chemotypes and *m*/*z* ion intensity to reference materials (Tikkisetty et al*.,* 2023a, b; Schmitz et al*.,* 2020). Additional related software allows heatmaps to work in tandem with multivariate statistical methods, such as the principal component analysis (PCA) and the discriminant analysis of principal component (DAPC) to visually examine clusters of chemotypically similar data. Samples can then be matched by a model created using reference sample data. In the case of plant-based oils, DART-TOFMS has been used to detect the adulteration of olive oils with hazelnut oil and identify the extraction method used to produce it (Vaclavik et al*.* 2009). More pertinent, success has been found using heatmaps generated from sample spectra collected by DART-TOFMS to create a library characterizing petroleum oils, diesel, diluted bitumen, fuel oils, and lubricating oils similar to the ForeST database for endangered wood species (Tikkisetty et al*.,* 2023a, b).

In this study, we applied Tikkisetty et al.’s methodology of analyzing petroleum oils to plant-based oils (Tikkisetty et al*.,* 2023a, b). Using DART-TOFMS to collect spectra of plant-based oils, the study aimed to fulfill three goals: firstly, to determine if the novel technique of using hydrophobic paper as the sampling method could successfully collect representative oil for forensic identification; secondly, to determine if the collected sample on hydrophobic paper would allow heat map generation for oil typing; and thirdly, to determine if the hydrophobic paper sample for unknown and weathered plant-based oils could be correctly typed and identified to their source oils using multivariate statistics.

## Materials and methods

### Reagents and sample preparation

Five plant-based oil types were analyzed in this study: avocado, canola, olive, peanut, and sesame. At a minimum, five different samples were used to represent each oil type, with details provided in SI Table S1. Oil samples were sourced locally from laboratory personnel and local grocery stores. Aliquots were taken from the retail container and stored in 40-mL amber borosilicate TraceClean® vials purchased from VWR. All samples were stored in a fridge at temperatures 5–9 °C. For DART-TOFMS analysis of source oils, aliquots were transferred to 10-mL or 20-mL clear, heat-treated borosilicate vials for ease of testing.

During preliminary testing, there was minimal sample preparation. For DART-TOFMS sampling, closed-end borosilicate capillary tubes from Fisher Scientific (Ottawa, Canada) were submerged in source oil sample aliquots for approximately 1 s. Excess oil was removed from the capillary tube using the lip of the borosilicate vial before being presented at the sampling gap. This was repeated a minimum of 5 times per sample. Data for these samples was used to prepare models for future application to blind quality assurance and weathered oil sample identification.

To evaluate the statistical model’s ability to type-match oils, six blind plant-based oil samples (within the stated categories) were prepared by a second analyst and supplied for quality assurance assessment. The unknown oils were stored under the same refrigeration conditions as the reference samples prior to analysis. Following statistical modeling creation, the acceptability of the procedure using hydrophobic paper sample collection, as intended for use in environmental spill situations, was evaluated using the procedures described in the following sections.

OmniSolv grade acetone and dichloromethane (DCM) used for rinsing glassware were purchased from VWR (Mississauga, Canada). Filtered seawater was obtained via an underground pump located at the Pacific and Environmental Science Center (PESC) and sourced from the Burrard Inlet (British Columbia, Canada).

### Weathered oils: microcosm preparation

Avocado, canola, olive, peanut, and sesame oil were chosen to observe weathering effects on the oil-type matching method. A single sample from each oil type was selected as a representative sample. To determine the representative sample of each type, leave-one-out cross-validation (LOOCV) scores for each sample and its replicates in a discriminant analysis of principal component (DAPC) model were examined; the sample of each type that displayed the highest and most consistent cross-validation scores within its replicates was chosen, as discussed later in this study. The 600-mL glass beaker microcosm containers were each pre-rinsed three times with acetone, followed by three times with DCM. Once the DCM had evaporated, 400 mL of seawater was added to the beakers. Each test oil sample (10 mL) was separately added to the surface of an individual seawater container using a 10-mL mechanical pipette, forming a surface oil slick. The weathering microcosm beakers were then placed inside a glass fish tank. A glass cover was placed on top of each tank leaving a 5-cm gap to allow for airflow across the surface while protecting the beakers from precipitation-caused disturbances. The weathering microcosms were placed on a wooden pallet under high cover for the duration of the experiment. An additional microcosm without oil was employed as baseline control.

The weathering was performed from December 18, 2023, to January 19, 2024. During this period, the average temperatures fluctuated between a high of 10 °C and a low of − 8 °C, as noted in Supplementary Information (SI) SI Table S2 (Government of Canada historical weather data. Accessed on Jan (2024)). The outdoor conditions exposed the microcosm to the West Coast winter weather conditions of British Columbia, Canada.

### Weathered oils: sampling procedure and microcosm conditions

Sampling began on December 22, 2023—the fifth day of the weathering experiment. The microcosms were brought inside the laboratory for sampling and were at room temperature for up to an hour. Whatman® phase separator filter papers obtained from Sigma-Aldrich were used to extract weathered oil samples (or blank control seawater) to explore the application of this hydrophobic paper for on-site sampling collection. Ziploc bags purchased from Amazon and 20-mL heat-treated borosilicate vials with PTFE-lined lids were used to store hydrophobic paper samples. Tweezers and scissors used to handle hydrophobic paper were rinsed in DCM prior to use.

Prior to sampling, a circular hydrophobic paper (Whatman® phase separator filter papers, Sigma-Aldrich) was cut in half and then cut into thin strips, approximately 35 mm in length and 4 mm in width. To begin sampling, tweezers were used to pick up a piece of hydrophobic paper and submerge it into the beaker perpendicular to the oil slick. After being submerged, the paper was dragged slightly back and forth for approximately 5 s before removing it from the beaker. This sampling was repeated ten times for each sample; five paper sample replicates were stored in Ziploc™ bags, and the remaining five were stored in capped 20-mL heat-treated borosilicate vials. The tweezers were then rinsed with DCM before extracting the next set of hydrophobic paper samples. Hydrophobic paper samples were stored for up to 4 weeks in a fridge at temperatures 5–9 °C prior to the DART-TOFMS analysis. Hydrophobic paper samples were collected at time points of 5-day, 12-day, 19-day, 26-day, and 33-day microcosm weathering, and details on sampling dates can be found in SI Tables S3, S4, S5, S6, and S7, respectively.

### DART-TOFMS parameters

The DART-SVP from IonSense (Saugus, MA USA) was the ion source, interfaced with the AccuTOF-DART 4G Mass Spectrometer from JEOL USA, Inc. (Peabody, MA USA). The DART-TOFMS was operated in positive ion mode, with a source heater temperature setting of 400 °C. The DART ion source was placed to allow a 2-cm sample gap between the insulator cap and orifice 1. Spectra were obtained over a range of 70–1000 m/*z* with each run containing five to six samples. Each run was a maximum of 30 min. Mass spectrometer settings are listed in SI Table S4.

### DART-TOFMS data acquisition

Polyethylene glycol 600 (PEG 600) from Tokyo Chemical Industry was used to calibrate the mass spectrometer. Closed-end borosilicate capillary tubes were used to introduce PEG 600 and oil samples for data acquisition and preliminary statistical model building. Whatman™ silicone-treated hydrophobic paper from Millipore Sigma (Oakville, Canada) replaced the capillary tubes for data collection of the weathered oil experiment. The runs included analysis of five replicate blanks of silicone-treated hydrophobic paper dipped in the weathered control seawater. Each analytical run began with collecting a PEG 600 spectrum, followed by the oil samples. A minimum of five spectra were collected for each oil sample. At the end of each oil sample analysis, a PEG 600 spectrum was collected to confirm calibration.

### Heatmap generation

For the five oil types selected in this study, a total of 39 plant-based oil samples were used to characterize oil types by heatmap as the first step. Heatmaps are two-dimensional graphs of spectral intensity from the ions detected by DART-TOFMS, which is suitable for the visual analysis of a large number of sample spectra. For this study, five replicate spectra were collected for each sample with 195 spectra in total. Sample spectra were extracted by the instrument msAxel@LP Data Processing software using a 0.1-min interval of the sample signal and background subtraction. Blank hydrophobic paper background subtraction was employed for the weathering portion of the study in order to compensate for any background responses unrelated to the oils.

### Statistical analysis

Statistical models for each oil type were created using Mass Mountaineer™ software. The ions chosen for model building were based on a heatmap and filtered by the software program. Principal component analysis (PCA) plots were constructed to display inherent similarities and differences between various plant-based source oil classes, as well as between weathered samples and sources. Based on the principal component analysis, a PCA scatterplot was constructed, and a discriminant analysis of principal component (DAPC) model was built to identify the classification of the weathered plant-based oil samples. The number of principal components (PCs) used to construct each model was optimized to maximize the external validation score and leave-one-out cross-validation (LOOCV) accuracy score for the corresponding DAPC.

## Results and discussion

### External validation of model using glass capillary tubes

**Fig. 1:**
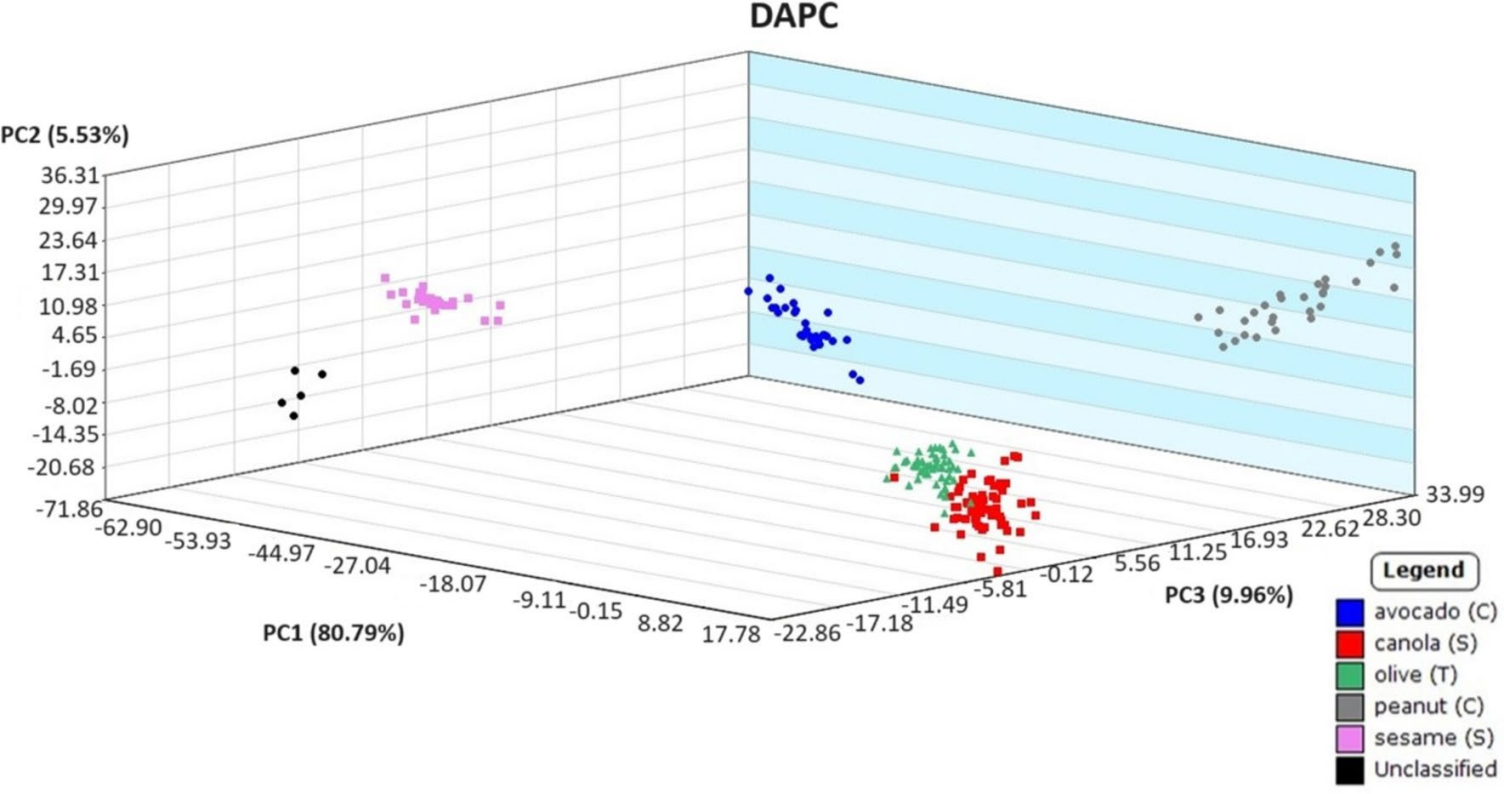
Discriminant analysis of principal component (DAPC) plot of source plant-based oils used to identify an unknown plant-based oil (in black)

Before exploring sampling with hydrophobic paper and analysis of weathered oil samples, a preliminary model was created using source oils sampled with borosilicate glass capillary tubes. This initial model was necessary to ensure plant-based oils were able to be correctly classified prior to proceeding with the proposed sampling technique. Three plant-based oil samples were prepared blind separately by a secondary analyst. These quality assurance samples were analyzed as “unclassified” samples, and the statistical model was applied for identification. The DAPC model generated from the data collected using the capillary tubes displayed distinct grouping between oil types while correctly matching unknown oils to their sources. In Fig. 1, all replicates of an unknown sesame oil sample were correctly identified, with a probability of up to 99.76%. Clearly, the unweathered vegetable oils could be categorized correctly using the statistical model, which led to the possibility of using hydrophobic paper to collect weathered oil in microcosms for identification.

### Determination of data consistency between the use of capillary tubes and hydrophobic paper for sample analysis

**Fig. 2:**
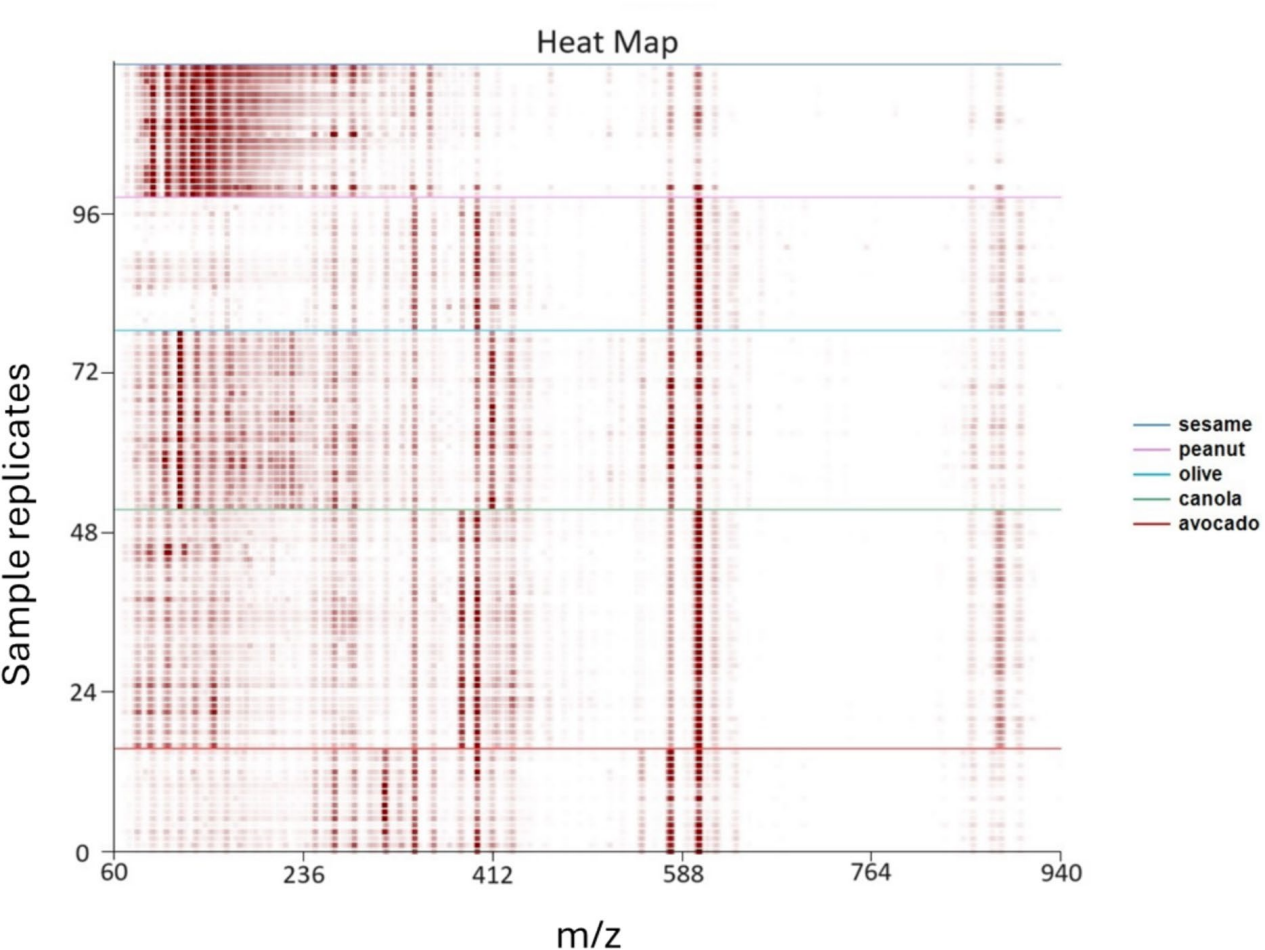
Heat map obtained using hydrophobic paper to introduce plant-based oil samples into the DART-TOFMS; top to bottom sesame, peanut, olive, canola, and avocado oil

The studied oils were compared by heatmap from samples analyzed by introduction to the DART using open-ended glass capillaries and hydrophobic paper (SI Fig. S1 and Fig. 2). For general information, each horizontal row (*y*-axis) of a heatmap displays a single sample spectrum. The *x*-axis displays the *m*/*z* ratio of the ion, with the range increasing from left to right. The abundance of each ion is shown through the shading of its point; more abundant ions appear darker in comparison to other ions. It is further noted that the intensity of this shading can be adjusted up or down overall for better visual viewing. The heatmap allows analysts to observe compositional trends between and within oil types. For the current study, it can be observed that the replicates all showed the same heatmap pattern with occasional differences in the intensity of ion signals.

The hydrophobic paper has been previously analyzed for its ability to separate oil and water in comparison to filtration methods (Ruan et al*.,* 2020). Building on that concept, this study compared its use as both a sampling device and an introduction vehicle against that of the standard method of using a glass capillary tube (Tikkisetty et al*.,* 2023a, b; Robert et al*.,* 2001; Vaclavik et al*.,* 2009). The generated heatmaps were found to be very similar despite the differing sampling techniques, with characteristic chemotypes being identified using both sampling techniques (SI Fig. S1 and Fig. 2). This result supported a primary study aimed to confirm if hydrophobic paper could be used for sample collection. The observed differing intensities, within oil types, of some ions could be explained by the natural variability occurring with hand sampling. It is recognized that DART-TOFMS is an exceptional technique in producing rapid real-time analysis (Huang et al*.,* 2011) but can show a variable ion relative intensity with each sampling (Price et al*.,* 2022). A representative sampling was obtained in practice by a collection of multiple replicate spectra from a single sample to achieve an average of the key oil chemotypes.

**Fig. 3:**
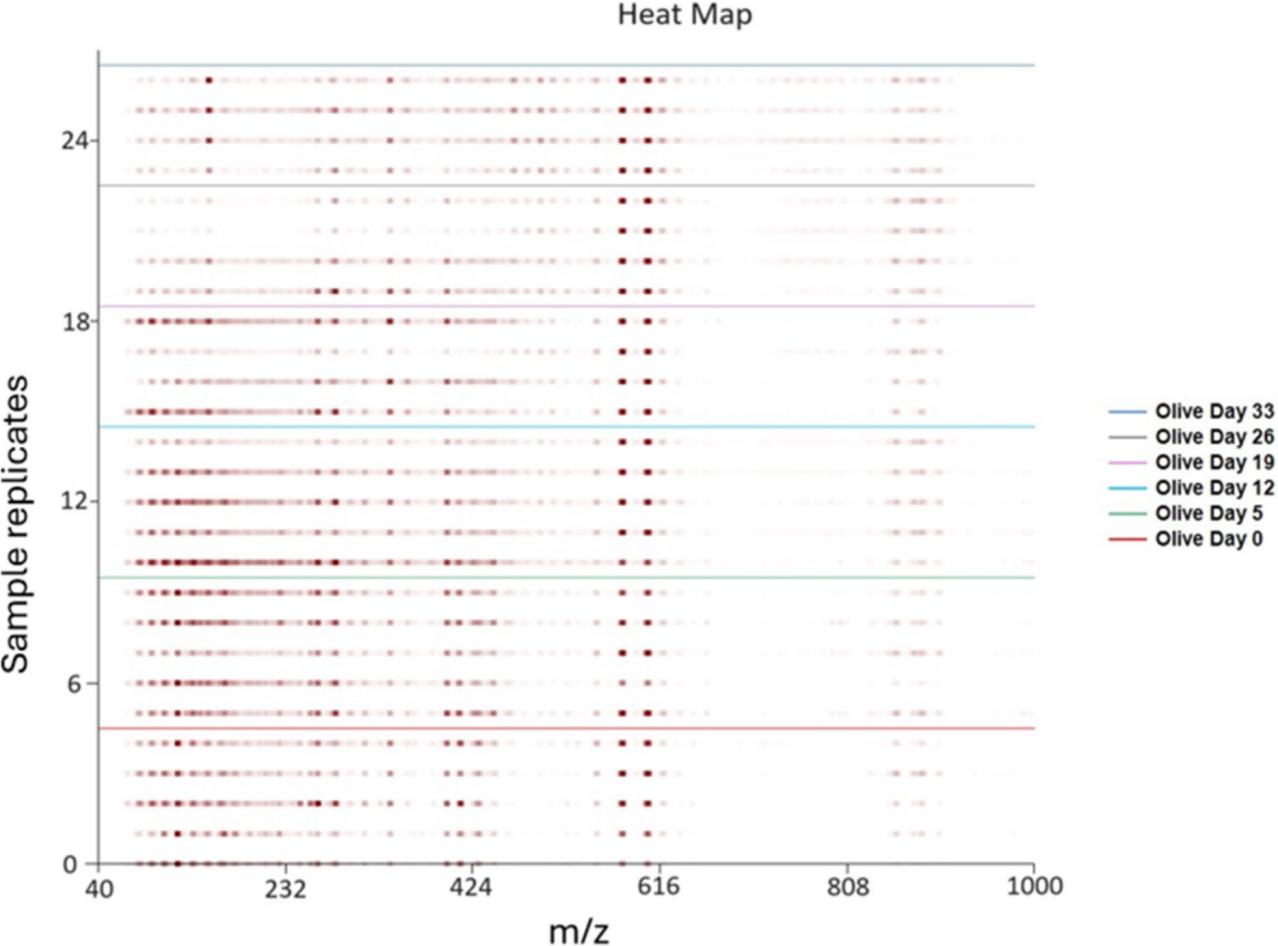
Heatmap showing DART-TOFMS responses for olive oil samples following storage in a Ziploc bag, with source oil (day 0) at the bottom and increasing in weathering period to day 33 at the top

Further to the primary aim, the potential of cross-contamination by leaching additives of plastic bags as a sample container was also investigated. The proposed sampling process consisted of collecting samples with hydrophobic paper, keeping the hydrophobic paper with sampled oil in a Ziploc™ bag for easy transportation, and storing the sample within the bag until analysis. The compounds such as slip agents and plasticizers from the plastic seeping into the oil sample were potential challenges (Christopher, et al*.* 2022) that required investigation. For comparison, plant-based oil samples were collected after 5, 12, 19, 26, and 33 days of weathering and stored in both a glass container and a Ziploc plastic bag in a fridge that ranged in temperatures from 5–9 °C. Heatmaps of olive oil sampled on each respective day were generated, allowing for a visual comparison of the two sample storage methods. The heatmaps generated are shown in SI Fig. S2 and Fig. 3, respectively.

In fact, heatmaps for olive oil samples stored in both a glass container (SI Fig. S2) and a Ziploc bag (Fig. 3) were visually similar. The same key characteristic ions were not lost in storage, and no contamination of plastic components from the bag can be seen in comparison to the equivalent sample stored in glass. The differing ion intensities appear to be unrelated to the container type. The noticed differences were attributed to the previously discussed hand sampling technique. These oil samples were stored at a temperature of 5 to 9 °C and not subjected to weathering, which is discussed later in this study.

Based on the presented comparable heatmap results, it was concluded that the sample collection method using hydrophobic paper produced positive results based on heatmap oil typing. Additionally, the ease of paper collection and storage in Ziploc bags for transport was a significant improvement to the collection procedure. To further investigate the effects of hydrophobic paper for oil sampling, the traditional storage method of glass vials was used to isolate the effects of hydrophobic paper.

### Comparison of petroleum oil to plant-based oil for distinguishing environmental spill

**Fig. 4:**
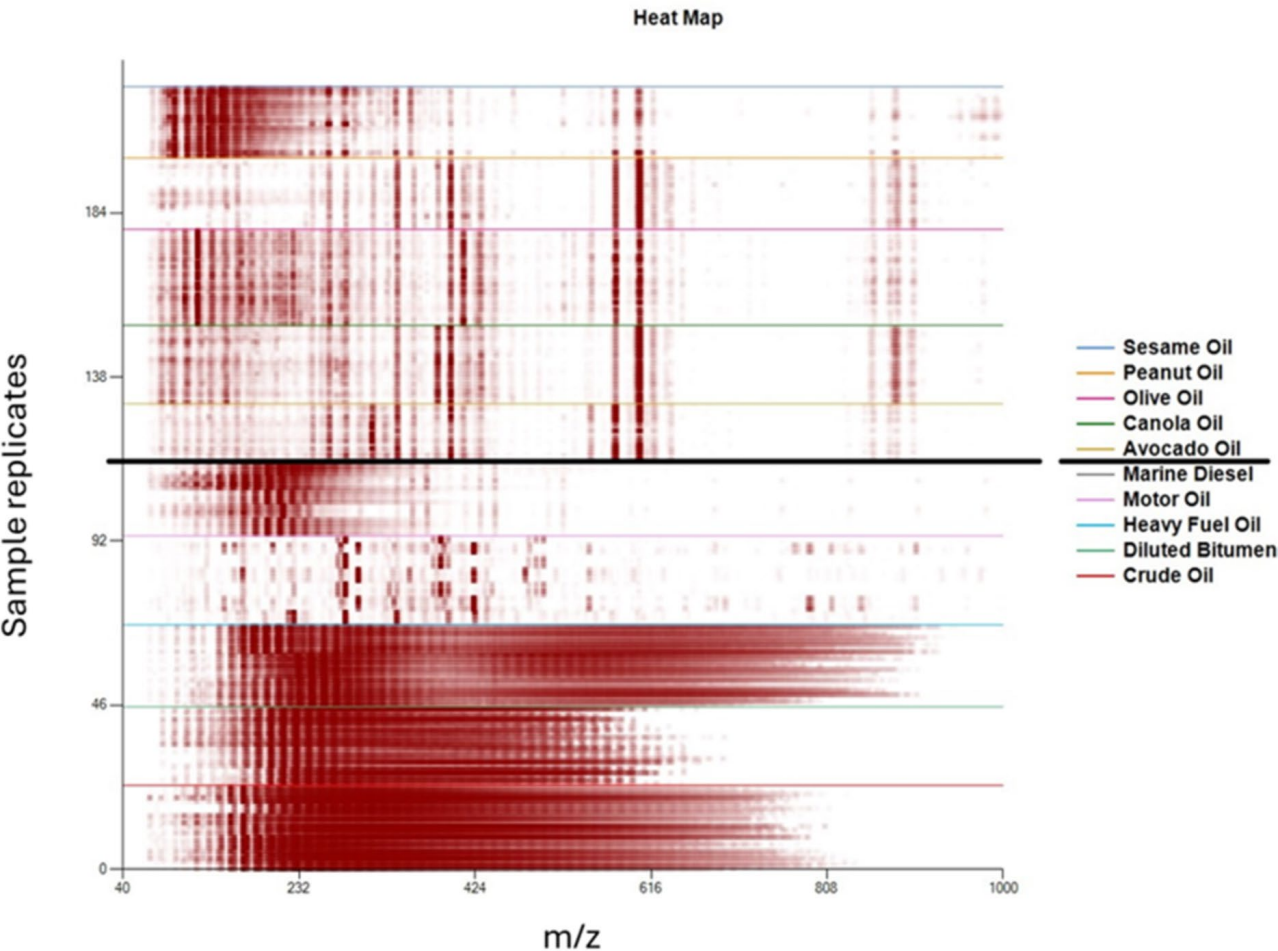
Heat map by DART-TOFMS of plant-based oils (from top, sesame, peanut, olive, canola, avocado) and petroleum oils (from center down; marine diesel, motor oil, heavy fuel oil, diluted bitumen, crude oil)

When oil spills occur in marine environments, it is crucial to determine the oil type to ensure that proper and effective clean-up measures are taken. Therefore, it is important to identify if the spilled oil is petroleum or plant-based. These two oils behave differently in marine environments and have differing responses to weathering processes (Fingas 2015). Unlike petroleum oils, plant-based oils do not readily evaporate, disperse, dissolve, or emulsify (Fingas, 2015). Some plant-based oils will solidify and polymerize when exposed to environmental media (Al-Darbin et al*.* 2005; Mudge, 1997; Salam et al*.,* 2012). These behaviors of plant-based oils make them harmful to marine animals and shoreline life (Fingas, 2015; Al-Darbin, et al*.* 2005; Salam et al*.,* 2012). Given these chemical and physical differences, it was hypothesized that plant oils and petroleum oils would be readily distinguishable from each other. The corresponding heatmaps of petroleum and plant-based oils are shown in Fig. 4.

**Fig. 5:**
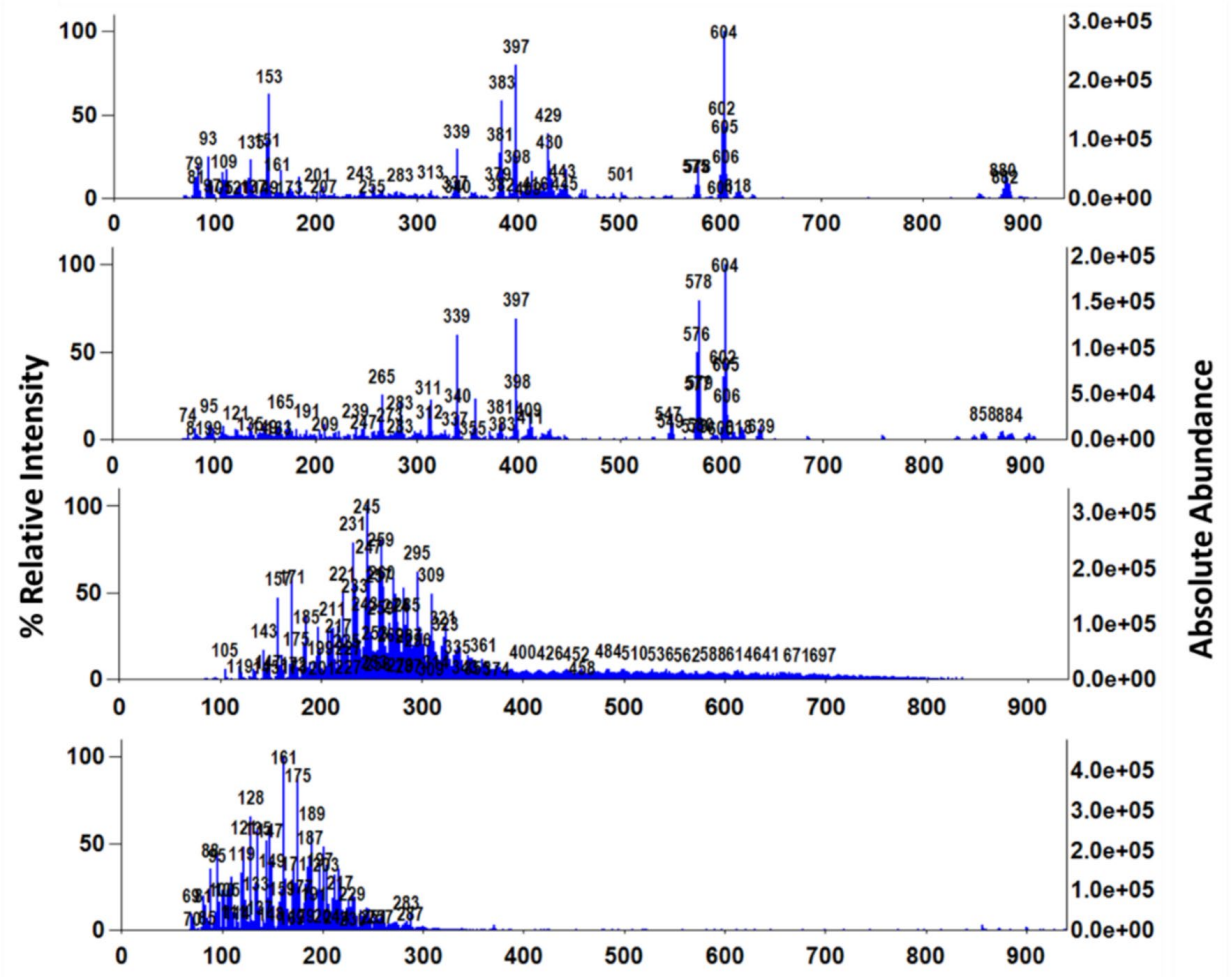
Comparison of DART-TOFMS mass spectra; top to bottom, for canola oil, avocado oil, bunker C oil, and marine diesel

Plant-based oils were observed to have more dispersed target ions compared to the dense signals from petroleum oils, particularly in the 192 to 900 m/*z* range (Fig. 4). The petroleum oils showed a repeating series of ions from such components as *n*-alkanes. DART-TOFMS mass spectra of plant oils were clearly visually distinct from the petroleum oils, observed by both individual mass spectra (Fig. 5) and multi-sample viewing by heatmap (Fig. 4). This is not surprising, given the chemical differences between the two. Petroleum oils are essentially a complex mixture of alkylated hydrocarbons and aromatics, while plant-based oils consist of fatty acids and their esters and mono-, di-, and triglycerides.

**Fig. 6:**
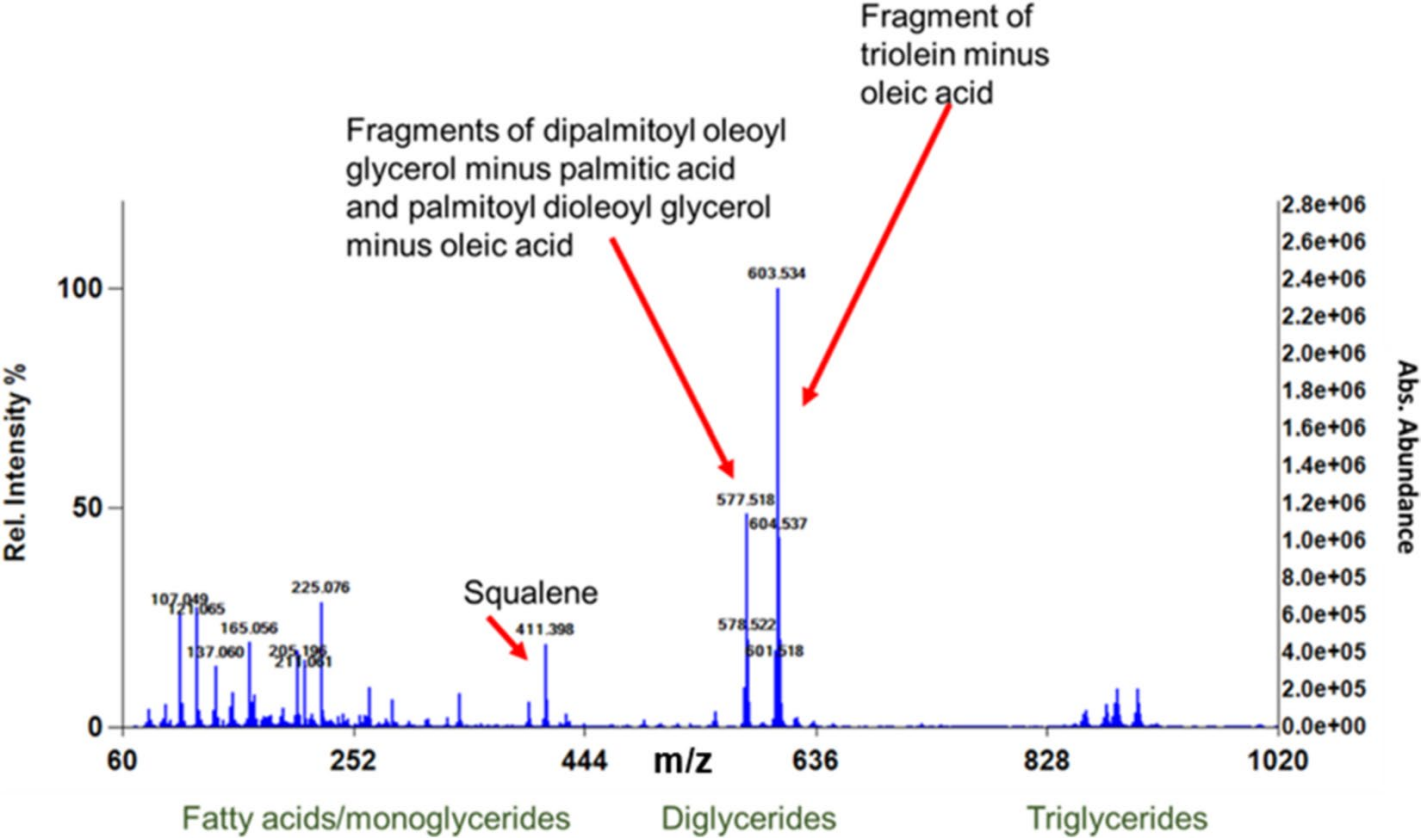
Olive oil DART-TOFMS mass spectra, showing annotation with tentative identification (Vaclavik et al., 2009)

An example of olive oil mass spectra (Fig. 6) displays monoglycerides in the region of 300–350 m/*z*, diglyceride fragments 500–600 m/*z*, and triglycerides around 800–900 m/*z*. These results corresponded well with those reported during a study of olive oil quality (Vaclavik et al., 2009). Based on the abundance of the observed key mass ions and the absence of ions due to the petroleum oil refining process, plant-based oils are readily distinguishable from petroleum oils by DART-TOFMS.

**Fig. 7:**
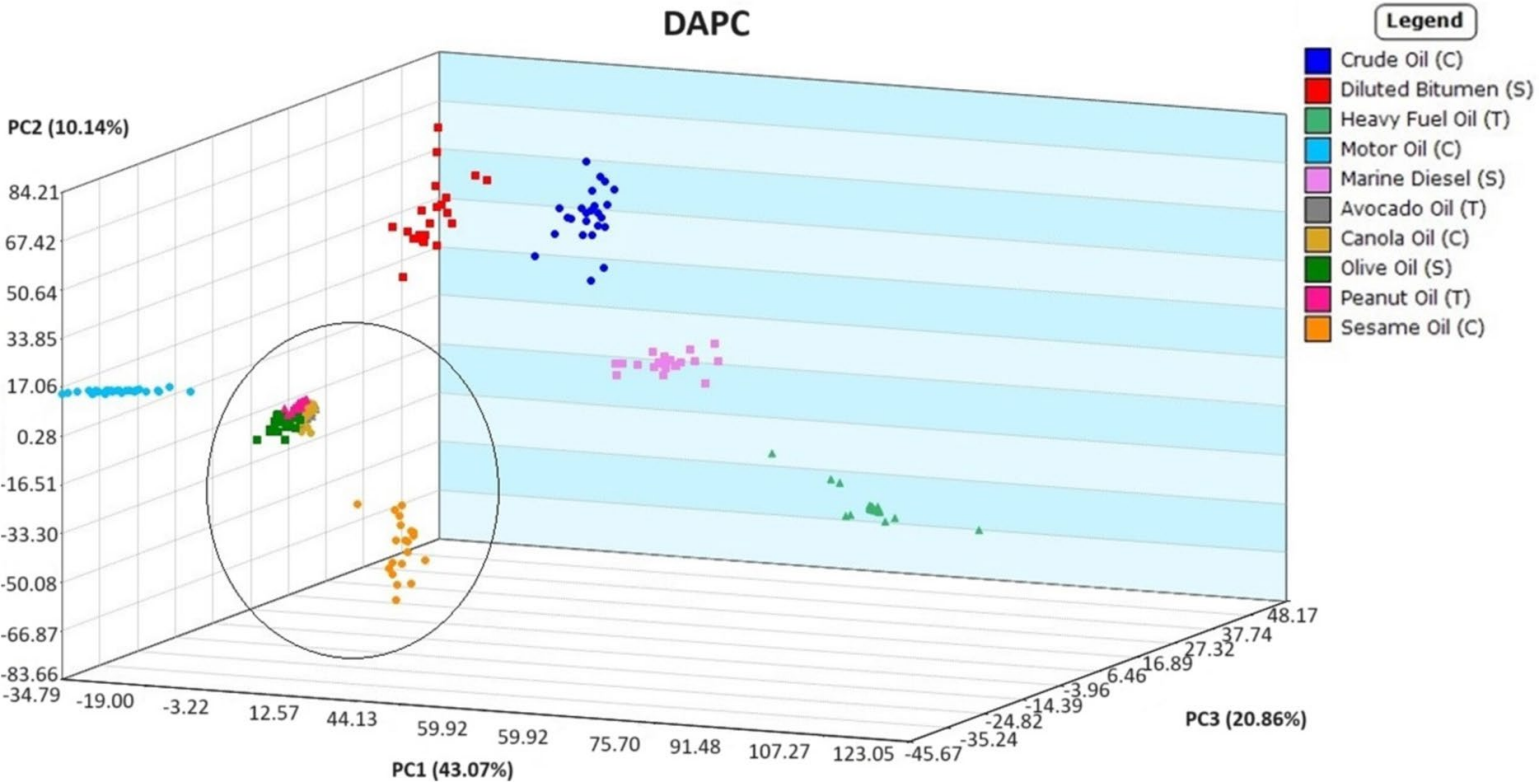
Discriminant analysis of principal component (DAPC) plot of DART-TOFMS results for all oil samples for comparison of petroleum versus plant-based oils (encircled for ease of viewing). Key top to bottom, crude oil, diluted bitumen, heavy fuel oil, motor oil, marine diesel, avocado oil, canola oil, olive oil, peanut oil, and sesame oil

While some of the vegetable oils were closer in visual fingerprint pattern than others, such as avocado and canola oil (Fig. 5), Mass Mountaineer software was able to sift through and select significant ions to distinguish each oil type and use the results to generate principal component analysis (PCA) scatterplots. PCA is a dimensionality reduction method for visual review that is not typically employed as a classification method (Tikkisetty et al*.,* 2023a, b), but discriminant analysis of principal component (DAPC) is an effective classification method (Jombart et al*.,* 2010). Principal components comprising at least 85%, but less than 100%, of the variance were used as the feature inputs for discriminant analysis. The DAPC method was applied to the same dataset to make a prediction model that can be seen in Fig. 7. Another directional view of this model can be found in SI Fig. S3. As suggested by the heatmap, there is a clear differentiation between petroleum and plant-based oils, providing a rapid confirmation of oil type in support of environmental mitigation measures and remediation processes. Plant-based oil samples, encircled in Fig. 7 (avocado oil hidden behind others), showed close clustering together, suggesting their composition was more similar to each other than the wider dispersed petroleum oils. This clustering was investigated further in order to determine if different types of plant-based oils could be distinguished from one another by the current method.

### Distinguishing plant-based oils between oil-type groups

**Fig. 8:**
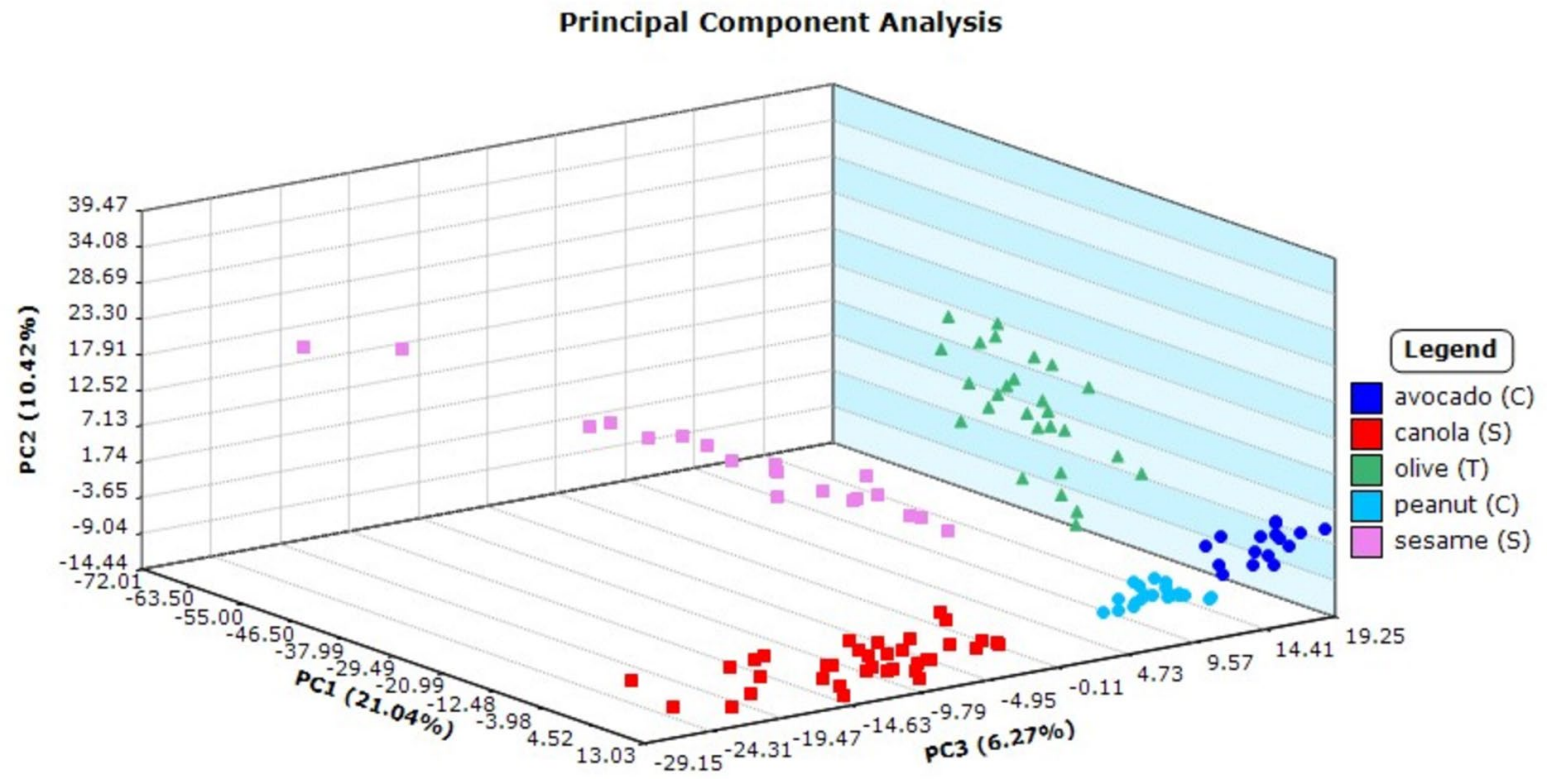
Principal component analysis plot of DART-TOFMS results for plant-based oils. Key top to bottom, avocado, canola, olive, peanut, and sesame

**Fig. 9:**
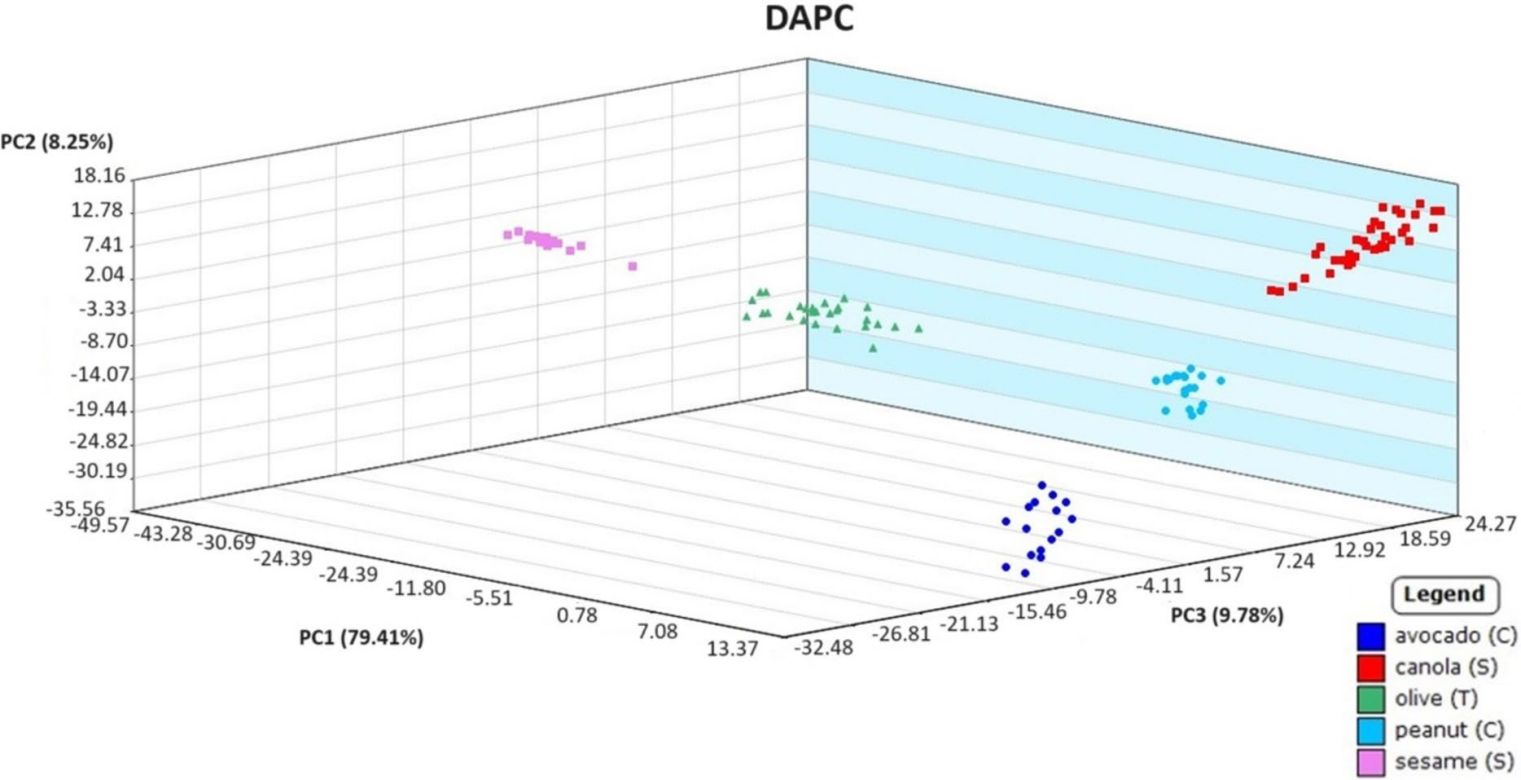
Discriminant analysis of principal component plot of DART-TOFMS results for plant-based oils. Key top to bottom, avocado, canola, olive, peanut, and sesame

Though all plant-based oils by heatmap showed very similar response ions, each of the oils is known to have differing physiochemical properties and logically were hypothesized to have differing characteristic spectral ion responses that could be exploited to distinguish the oils from one another. Furthermore, different carbon numbers and degrees of unsaturation in the fatty acid content of triglycerides, refining processes, and additives all contribute to the oil’s unique chemical fingerprint. Using the same mass ions selected to calculate the PCA scatterplot of the plant-based oils (Fig. 8), DAPC statistical analysis by the Mass Mountaineer® program was applied with the result shown in Fig. 9. In comparison to the petroleum oils, the spread of points “between” each plant-based oil group became distinct. The observed spread of points “within” a plant group may be attributed to variability in analyst sampling. Overall, there was a definitive separation between all five plant-based oil types, allowing each group to be visually distinguished from one another. To confirm the model’s accuracy, Mass Mountaineer software’s leave-one-out cross-validation (LOOCV) was applied. This internal validation automatically iteratively removes each spectrum from the training set of ions and checks its assignment based on the revised model. A view of the three-dimensional DAPC from other dimensional angles can be seen in SI Fig. S4.

### Evaluating classification of plant-based oils in comparison to petroleum oils following weathering processes

**Fig. 10:**
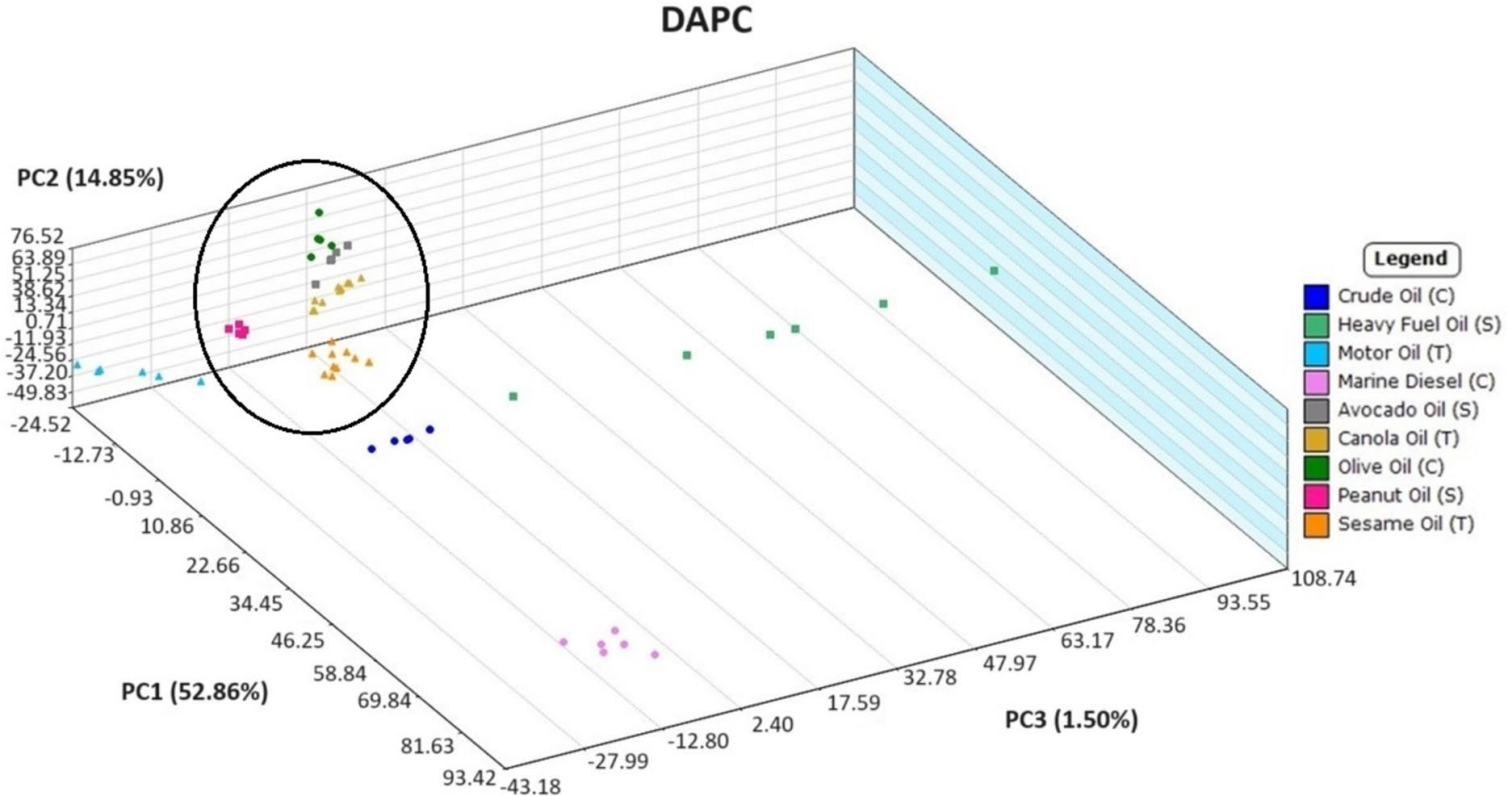
Generated DAPC of DART-TOFMS results for plant-based oils (encircled for ease of viewing) after 5 days of weathering and petroleum-based oils after 8 days of weathering. Key top to bottom, crude oil, heavy fuel oil, motor oil, marine diesel, avocado oil, canola oil, olive oil, peanut oil, and sesame oil

The method’s ability to distinguish petroleum and plant-based oils from one another following weathering was evaluated to determine its potential use in rapid oil spill identification. The plant-based oil samples were collected on day 5 of weathering, and petroleum oil samples were collected on day 8 of weathering. Though collected 3 days apart, the samples were exposed to the same environmental weathering as each microcosm setup on different days. The petroleum oil microcosm was set up exactly as the plant-based microcosm described in the “Materials and methods” section. DAPC of the weathered oils can be seen in Fig. 10 with other views of the DAPC seen in SI Fig. S5 and S6. As observed when analyzing the source oils to one another, the plant-based oil groups were relatively close together in comparison to the petroleum oil groups. This is expected because of the substantial differences in chemotypes between these oil types.

### Determining the stability of weathered plant-based oils

To evaluate weathering effects on the analyzed plant-based oil samples, a weathering microcosm was used to expose the oils to seawater in an outdoor environment. The weathered oil samples were collected 5, 12, 19, 26, and 33 days post exposure and analyzed to determine characteristic ions by DART-TOFMS analysis. Previous studies employing fatty acid analysis suggested that olive oil and sesame oil were the most and least stable plant-based oils, respectively (Orsavova, et al*.* 2015, Fingas, 2014). Thus, to investigate the statistical model’s ability to distinguish and type-match degraded oils, olive oil and sesame oil weathered samples were chosen for further study. The additional weathering analysis of avocado, canola, and peanut oils can be found in SI Figs. S7 – S20, respectively.

**Fig. 11:**
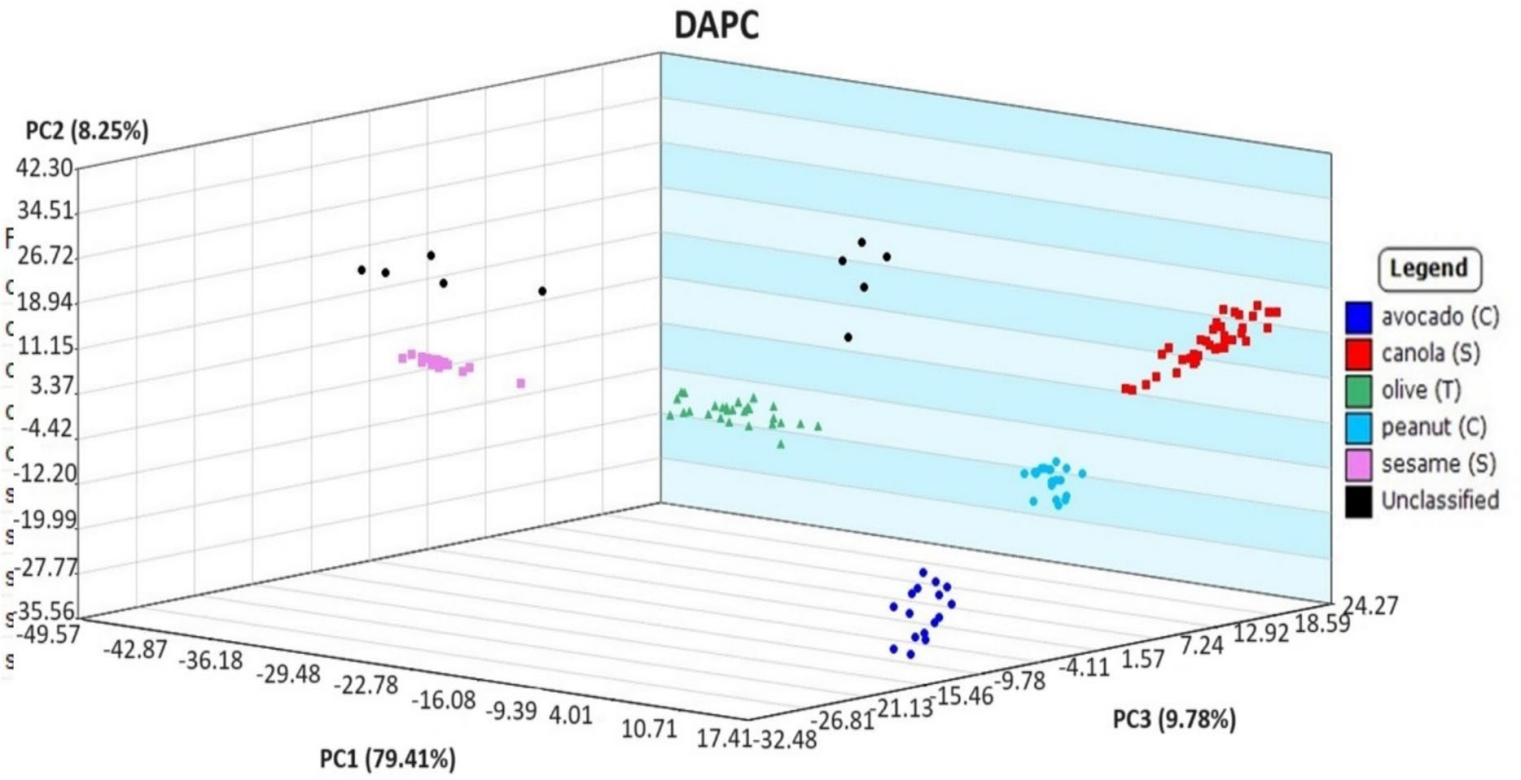
Discriminant analysis of principal component (DAPC) plot of DART-TOFMS results for plant-based oils comparing olive and sesame oil (unclassified) test samples after 5 days of weathering. Key top to bottom, avocado oil, canola oil, olive oil, peanut oil, sesame oil, and unknown oils

**Fig. 12:**
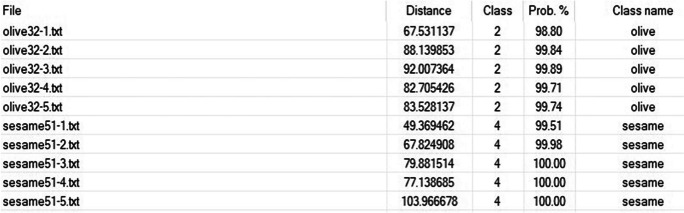
Classification probability results for Fig. 11 DAPC application showing the ten “unclassified” test samples, each after 5 days of weathering processes

Collected data for the 5-day weathered oils were all identified to their correct class with high probability, with examples given for sesame and olive oils in Figs. 11 and 12 (weathered oils as “unclassified” in the plot). There was a clear distinction between group types on the generated heatmap on day 5 with a relationship to their original source oils collected on day 0 of analysis. Surprisingly, the results of this study demonstrated canola and peanut oil to be the most resistant to weathering processes rather than olive oils. In previous studies, the percentage of oleic acid in canola oil and peanut oil has been found to be approximately 60% and 71%, respectively (Orsavova et al*.,* 2015; Fingas, 2014). Oils with a higher oleic acid content were more stable and resistant to weathering (Tamothran et al., 2022; Al-Darbin et al*.,* 2005). In addition to their high percentages of more oxidative stable fatty acids, canola oil and peanut oil also have low percentages of linoleic acid. Although the plant-based oils were previously reported as less stable due to their differing fatty acid content, the DART-TOFMS method in the present study was still able to match all of the 5-day weathered oils to their source oil. This result is supported by the knowledge that plant-based oils exposed to marine environments do not evaporate or spread like petroleum oils and experience fewer biodegrading processes that would alter their chemo-typing profile (Fingas, 2015).

**Fig. 13:**
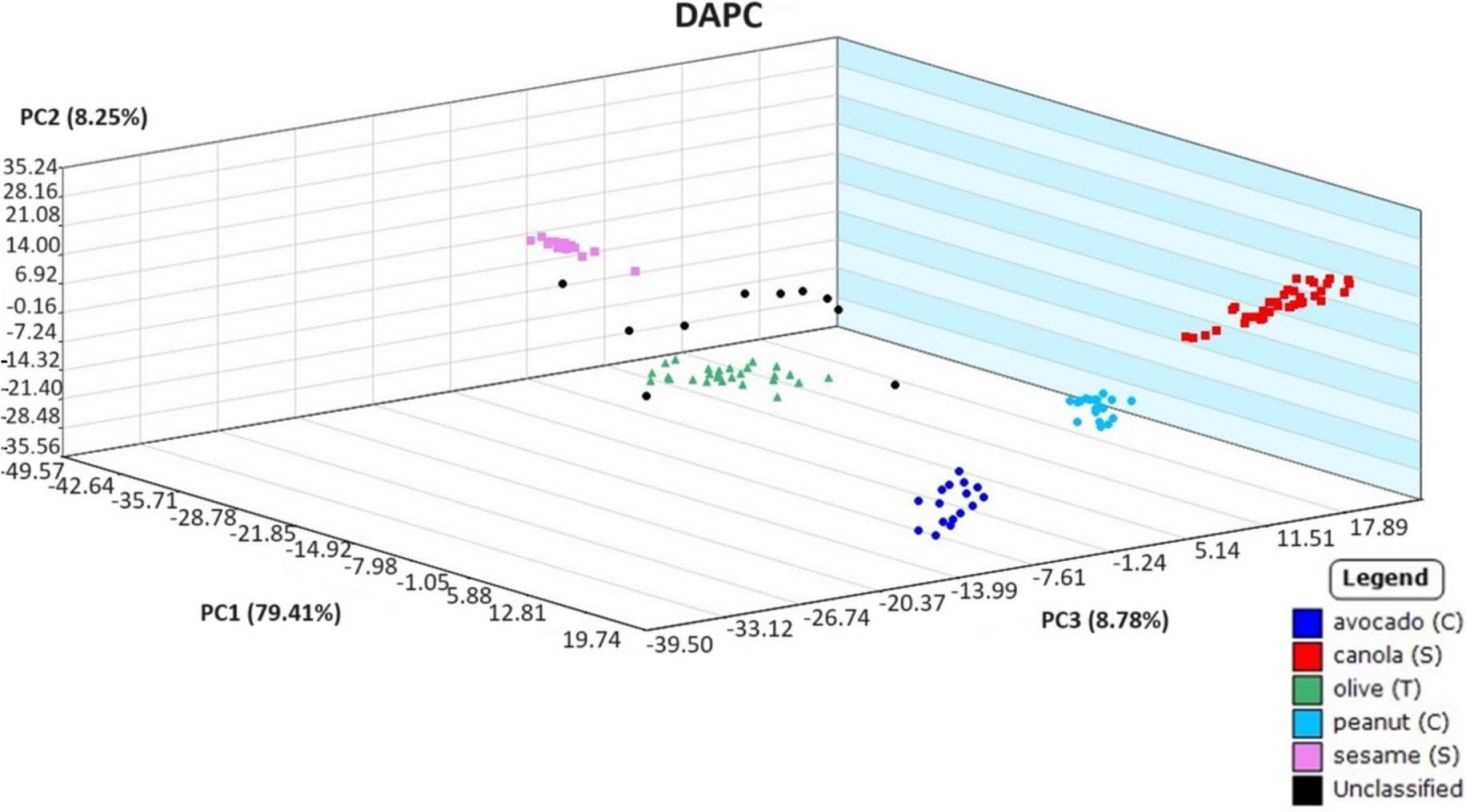
Generated heatmap of DART-TOFMS results for sesame oil day 0 (pink) and olive oil day 0 (green) with ten “unclassified” test samples. The test samples being five of each oil type following 12 days of the weathering process. Key top to bottom, avocado oil, canola oil, olive oil, peanut oil, sesame oil*,* and unknown oils

More extensive weathering of the unknown oil samples over 12 days and 26 days visually showed gradual scattered data points. When compared to 5 days of weathering (Fig. 11), the unknown samples seen in Fig. 13 as black dots do not form a distinct clustering. After 12 days of weathering, the generated DAPC (Fig. 13) showed an identifiable distinction between group types, while the grouping of the weathered samples showed more separation than that of 5 days. All ten of the 12-day weathered samples were classified to their source oil correctly with continued high probability. The generated DAPC of 26-day weathered oil samples showed significant spreading of the oil samples from their source. Clearly, the weathering processes led to the gradual increasing loss of characteristic compounds which were important for the forensic study. All ten of the 26-day weathered samples were classified incorrectly and not identified according to their source oil. Based on these results, this current DART-TOFMS method would be accurate for short-term weathered oil spills up to 12 days of exposure. Despite restrictions to the timeline of the microcosm experiment, the results of this study showed an invaluable contribution of this methodology to identify short-term weathered plant-based oil spills in a marine environment.

For clarity of the differences between DART-TOFMS and GC–MS, the latter includes chromatography to separate the responding biomarkers ions. This is obviously advantageous for specificity but equally is a very time-consuming process when oil spill mitigation requires an urgent answer to spill source and oil type. While DART-TOFMS does not attain this level of specificity, its biomarker mass spectrum is fast to collect and readily assessed by machine learning algorithms. Even including multiple potential spill sources in the analysis, results can be achieved within a day with DART/TOF analysis. Furthermore, as the current study results indicate, data from many oil samples can be collated into statistical models and form part of a library that can easily be scanned by the software for comparison and identification of an unknown sample. This approach is already being used in multiple other areas of study, such as the identification of endangered timber species in the fight against organized illegal logging (Espinoza et al*.,* 2021).

## Conclusion

The current DART-TOFMS method was shown to be successful at rapidly distinguishing plant-based and petroleum-based oil spills. While GC-FID and GC–MS procedures established by the European Standardization of oil spill identification are the gold standard, the procedure is time-consuming and labor-intensive. Further, the field sampling technique with glass containers is cumbersome and costly when dealing with a large scale of plant-based oil spill. In this current study, an alternative approach was proposed with the use of the rapid DART-TOFMS analysis in tandem with the simplified field sample collection using hydrophobic paper. A source oil model was developed with oil introduced to the DART-TOFMS by glass capillary, while weathered oils were collected on hydrophobic paper, stored in plastic bags, and analyzed directly without sample preparation in the laboratory. The DART-TOFMS analysis, including any additional reference oils required, took less than a day. The results highlight the method’s ability to enable easy paper collection of oil samples, rapidly type-match plant-based oils, and successfully classify them to their original source oil with high confidence. The procedure was further proven applicable to short-term weathered plant-based oil samples. The novelty of this methodology enhances the current sampling and analysis procedure for plant-based oil spill forensics and may hold a similar application to weathered petroleum oil identification. It is also recognized that the simple DART-TOFMS mass spectra can be collated in the form of a library for future reference, making analysis time even shorter and allowing data to be shared between laboratories.

## Data availability 

The datasets generated during and/or analyzed during the current study are available from the corresponding author on reasonable request.

## Supplementary Information

Below is the link to the electronic supplementary material.

Supplementary file1 (DOCX 3919 KB)
