# Supplementary material for: Identification of plant-based spilled oils using direct analysis in real-time–time-of-flight mass spectrometry with hydrophobic paper sampling
Source: Environ Monit Assess. 2025 Jan 14;197(2):171. doi: 10.1007/s10661-024-13583-1 (PMC11732964; doi:10.1007/s10661-024-13583-1)
Supplement: Supplementary file 1 — Supplementary file1 (DOCX 3919 KB) [file 10661_2024_13583_MOESM1_ESM.docx]

Identification of Plant-based Spilled Oils using Direct Analysis in Real Time Time-of-Flight Mass Spectrometry with Hydrophobic Paper Sampling

Paige McCallum ^a^, Genesis Saturos ^a^, Lola Rabinovitch ^a^, Taylor Filewood ^a^, Honoria Kwok ^a^, Jeffrey Yan ^a^, Robert Cody ^b^, Pamela Brunswick ^a^, and Dayue Shang ^a*^

a. Science and Technology Branch, Pacific Environmental Science Centre, Environment and Climate Change Canada, Pacific and Yukon Laboratory for Environmental Testing, North Vancouver, B.C., Canada.

b. JEOL USA Inc, Peabody, Massachusetts, United States of America

* Corresponding authors Dayue Shang [Dayue.Shang@ec.gc.ca](mailto:Dayue.Shang@ec.gc.ca) and [Pamela.brunswick@ec.gc.ca](mailto:Pamela.brunswick@ec.gc.ca)

| **Sample** | **Brand** | **Received** |
| --- | --- | --- |
| Olive 5 | Bisnak | 11/01/2023 |
| Olive 6 | Terra Delyssa™ | 11/3/2023 |
| Olive 7 | Sunshine Coast | 11/7/2023 |
| Olive 10 | Terra Delyssa™ | 11/7/2023 |
| Olive 23 | Lefas | 11/14/2023 |
| Olive 24 | Bertolli™ | 11/14/2023 |
| Olive 31 | Bertolli™ | 11/8/2023 |
| Olive 32 | Terra Delyssa™ | 11/8/2023 |
| Olive 48 | Filippo Berrio™ | 11/15/2023 |
| Olive 57 | No Name (N/A) | 11/15/2023 |
| Canola 11 | No Name (N/A) | 11/06/2023 |
| Canola 12 | No Name (N/A) | 11/03/2023 |
| Canola 22 | Sunfrie | 11/7/2023 |
| Canola 25 | No Name (N/A) | 11/14/2023 |
| Canola 38 | Sunfrie | 11/8/2023 |
| Canola 42 | President's Choice™ | 11/15/2023 |
| Canola 43 | No Name (N/A) | 11/15/2023 |
| Canola 45 | Harvest | 11/15/2023 |
| Canola 46 | PC Organics™ | 11/15/2023 |
| Canola 49 | No Name (N/A) | 11/15/2023 |
| Canola 54 | Crisco™ | 11/15/2023 |
| Canola 55 | Mazola™ | 11/15/2023 |
| Avocado 9 | Chosen Foods™ | 11/7/2023 |
| Avocado 14 | Chosen Foods™ | 11/7/2023 |
| Avocado 16 | Chosen Foods™ | 11/27/2023 |
| Avocado 20 | Harvest Peak | 11/7/2023 |
| Avocado 36 | President's Choice ™ | 11/8/2023 |
| Avocado 53 | Chosen Foods™ | 11/15/2023 |
| Sesame 19 | Foreway™ | 11/9/2023 |
| Sesame 35 | Lee Kum Lee™ | 11/8/2023 |
| Sesame 50 | Kadoya™ | 11/15/2023 |
| Sesame 51 | Great Value™ | 11/15/2023 |
| Sesame 58 | Dabur™ | 11/15/2023 |
| Peanut 21 | Saporita™ | 11/7/2023 |
| Peanut 63 | Compliments | 12/4/2023 |
| Peanut 64 | Planters™ | 12/2/2023 |
| Peanut 65 | Lion & Globe™ | 12/2/2023 |
| Peanut 69 | Great Value™ | 12/6/2023 |
| Peanut 70 | No Name (N/A) | 12/12/2023 |

**Table S1** Details on oil sample type used to create model.

| **Study Period** | **Date** | **Average Temperature** |
| --- | --- | --- |
| Day 1 | December 18^th^, 2023 | 8.4°C |
| Day 2 | December 19^th^, 2023 | 9.6°C |
| Day 3 | December 20^th^, 2023 | 9.9°C |
| Day 4 | December 21^st^, 2023 | 9.6°C |
| Day 5 | December 22^nd^, 2023 | 8.2°C |
| Day 6 | December 23^rd^, 2023 | 4.5°C |
| Day 7 | December 24^th^, 2023 | 8.2°C |
| Day 8 | December 25^th^, 2023 | 8.4°C |
| Day 9 | December 26^th^, 2023 | 9.3°C |
| Day 10 | December 27^th^, 2023 | 10.0°C |
| Day 11 | December 28^th^, 2023 | 10.4°C |
| Day 12 | December 29^th^, 2023 | 10.7°C |
| Day 13 | December 30^th^, 2023 | 10.2°C |
| Day 14 | December 31^st^, 2023 | 8.8°C |
| Day 15 | January 1^st^, 2024 | 6.2°C |
| Day 16 | January 2^nd^, 2024 | 7.2°C |
| Day 17 | January 3^rd^, 2024 | 8.3°C |
| Day 18 | January 4^th^, 2024 | 7.3°C |
| Day 19 | January 5^th^, 2024 | 6.8°C |
| Day 20 | January 6^th^, 2024 | 4.7°C |
| Day 21 | January 7^th^, 2024 | 3.1°C |
| Day 22 | January 8^th^, 2024 | 3.7°C |
| Day 23 | January 9^th^, 2024 | 7.0°C |
| Day 24 | January 10^th^, 2024 | 4.7°C |
| Day 25 | January 11^th^, 2024 | 1.0°C |
| Day 26 | January 12^th^, 2024 | -8.0°C |
| Day 27 | January 13^th^, 2024 | -6.8°C |
| Day 28 | January 14^th^, 2024 | -3.7°C |
| Day 29 | January 15^th^, 2024 | -1.2°C |
| Day 30 | January 16^th^, 2024 | 0°C |
| Day 31 | January 17^th^, 2024 | 0°C |
| Day 32 | January 18^th^, 2024 | 0°C |
| Day 33 | January 19^th^, 2024 | 2°C |

**Table S2** Average temperatures on North Vancouver, B.C., Canada during the microcosm study.

| Sample | Sample date Weathering | | | | |
| --- | --- | --- | --- | --- | --- |
|  | Day 5 | Day 12 | Day 19 | Day 26 | Day 33 |
| Avocado 14 | 12/22/2023 | 12/29/2023 | 01/05/2024 | 01/12/2024 | 01/19/2024 |
| Canola 49 | 12/22/2023 | 12/29/2023 | 01/05/2024 | 01/12/2024 | 01/19/2024 |
| Olive 32 | 12/22/2023 | 12/29/2023 | 01/05/2024 | 01/12/2024 | 01/19/2024 |
| Peanut 63 | 12/22/2023 | 12/29/2023 | 01/05/2024 | 01/12/2024 | 01/19/2024 |
| Sesame 51 | 12/22/2023 | 12/29/2023 | 01/05/2024 | 01/12/2024 | 01/19/2024 |

**Table S3** Days of weathered oil samples with sampling dates.

| **Parameter** | **Value** |
| --- | --- |
| Orifice 1 Voltage | 20V |
| Orifice 1 Temperature | 120°C |
| Detector Voltage | 2300V |
| Ring Lens Voltage | 5V |
| Orifice 2 Voltage | 5V |
| Ion Guide RF Voltage | 500V |

**Table S4** AccuTOF-DART mass spectrometer parameters.


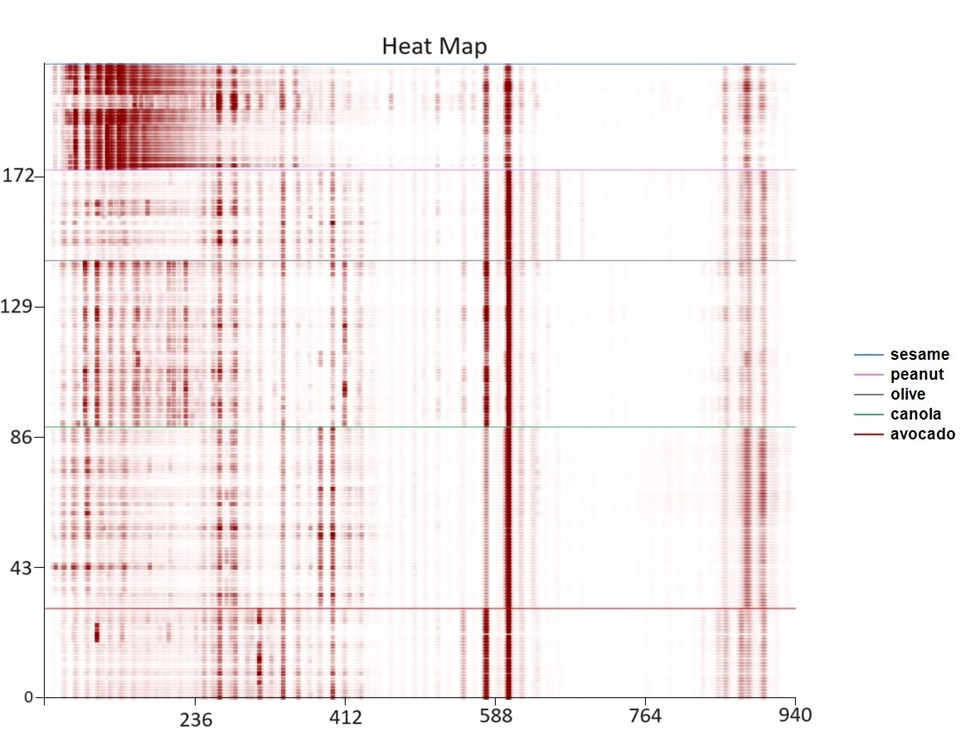


**Figure S1** Heat map using glass capillary tubes to introduce plant-based oil sample to DART.


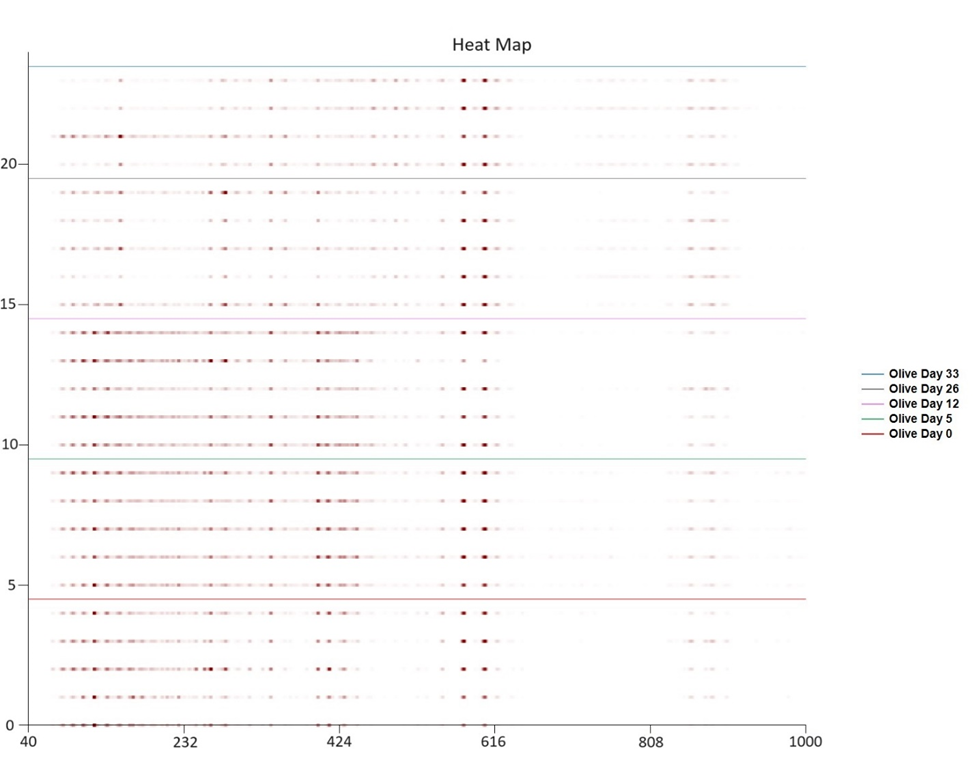


**Figure S2** Olive oil samples stored in glass container with PTFE lined lid, with source (day zero) at bottom and increasing in weathering period to day thirty-three at top.


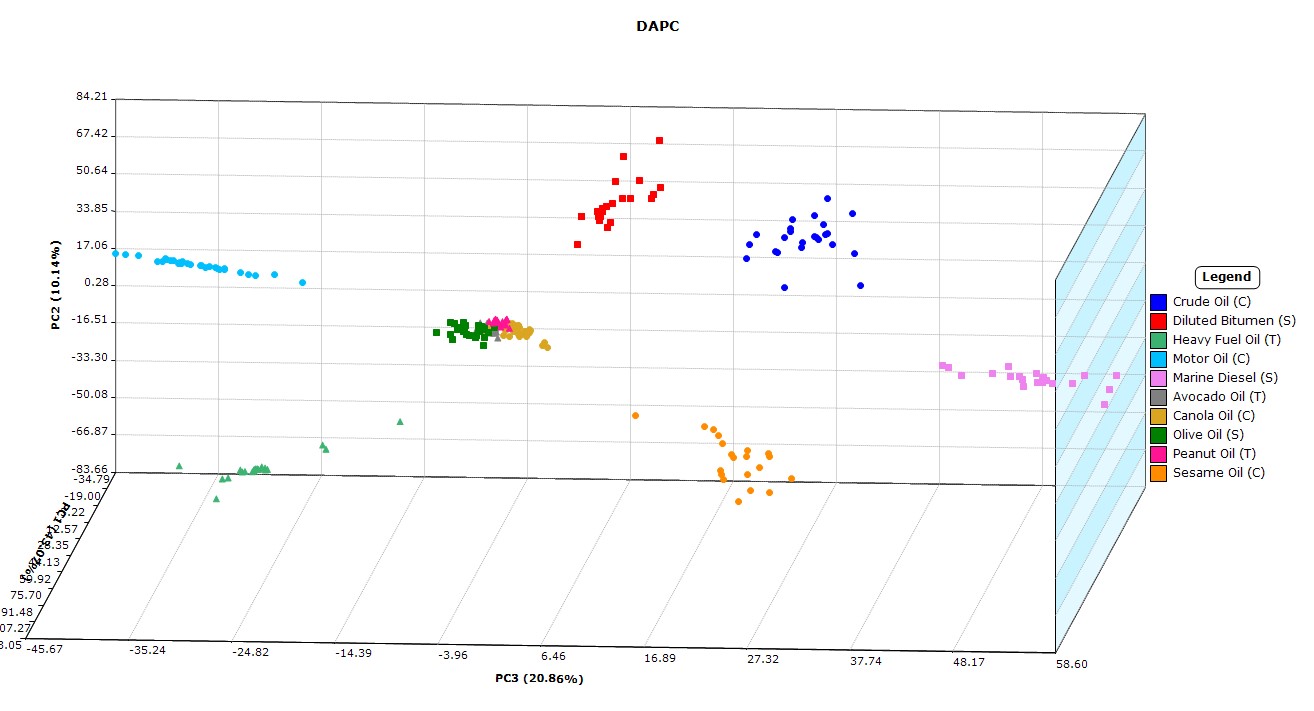


**Figure S3** Different view of Discriminative Analysis of Principal Components scatter plot of all oil samples.


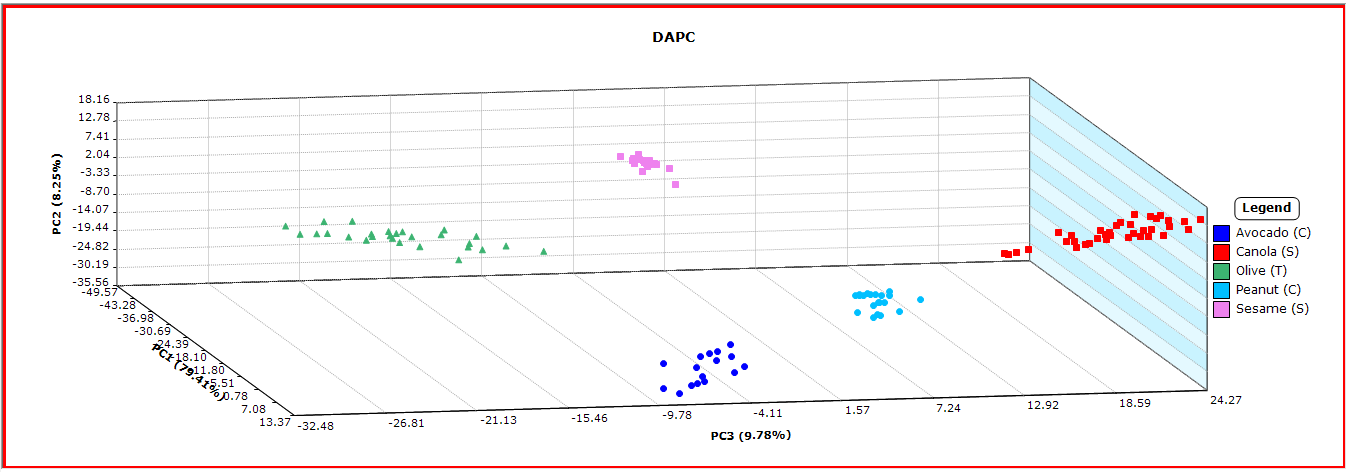
**Figure S4** Second view of Discriminative Analysis of Principal Components scatter plot of plant-based oils.

.


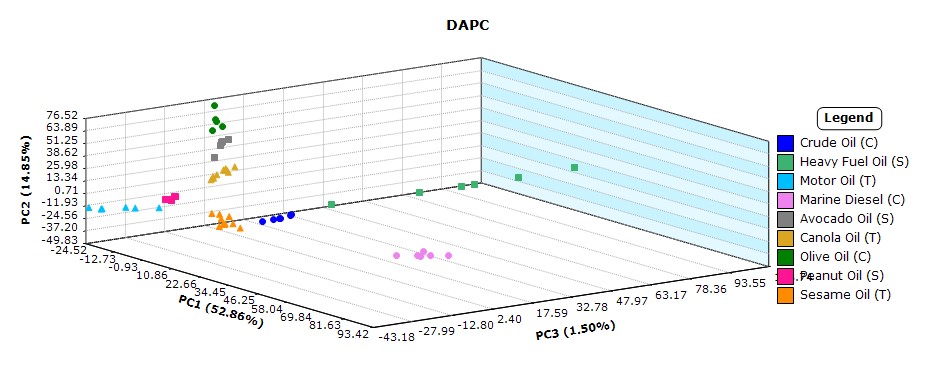


**Figure S5** Generated DAPC of plant-based oils, day five of weathering and petroleum-based oils, day eight of weathering for classification. Second view.


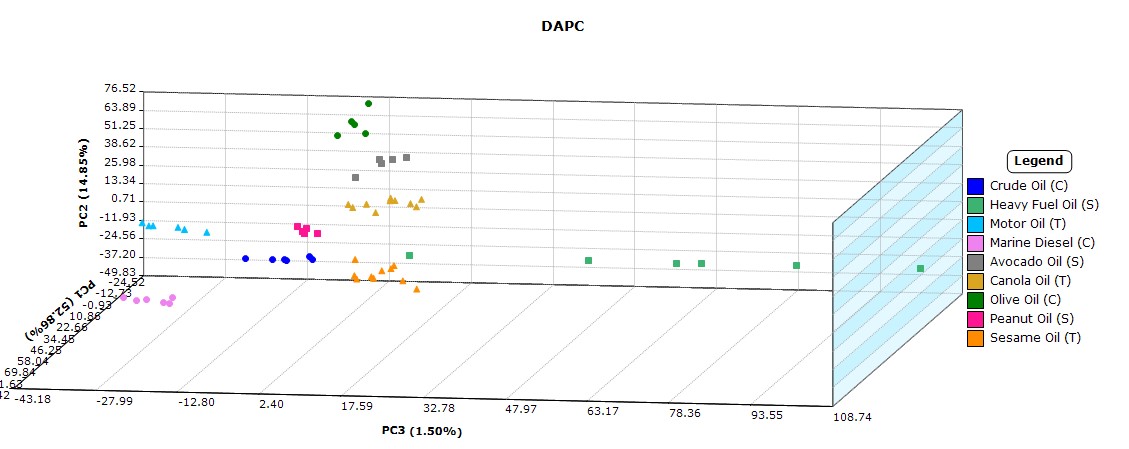


**Figure S6** Generated DAPC of plant-based oils, day five of weathering and petroleum-based oils, day eight of weathering for classification. Third view.


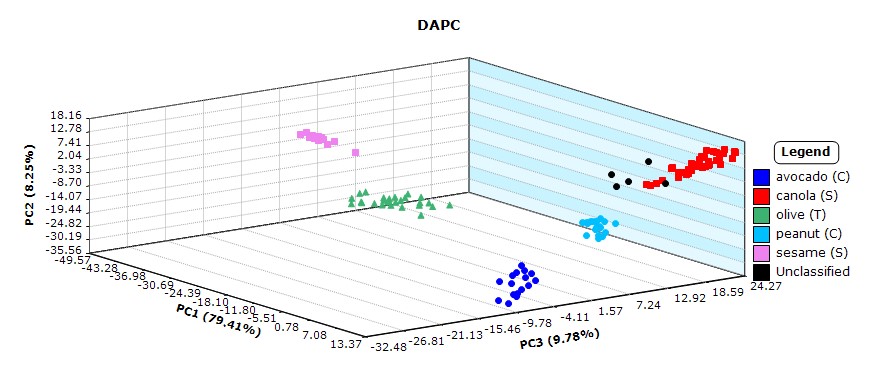

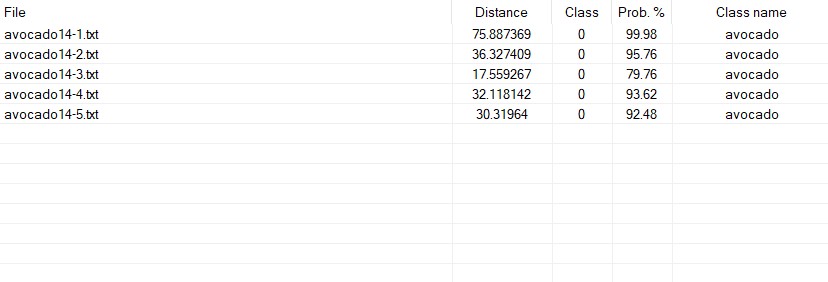


**Figure S7** Avocado oil weathered on day 5 classifying correctly.


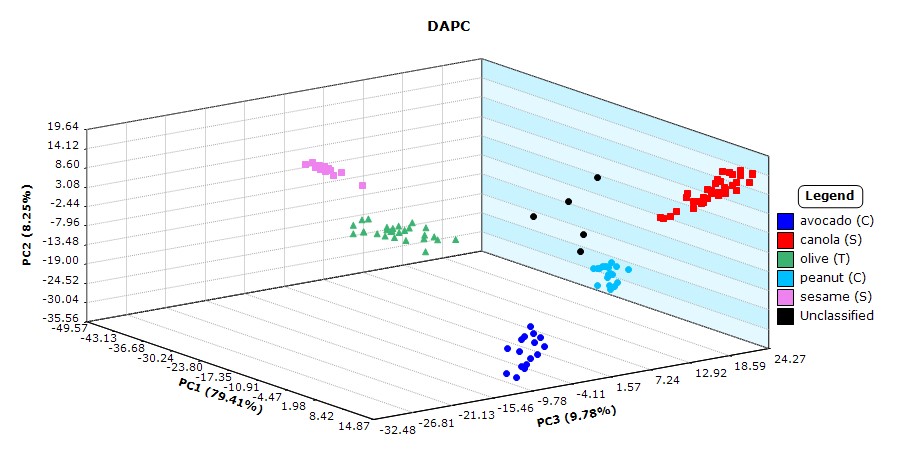
*
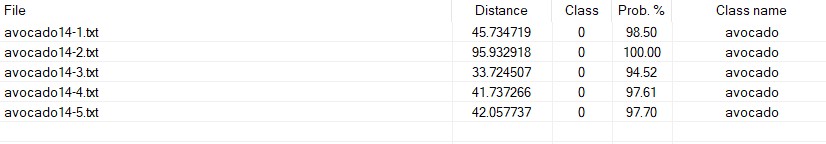
*

**Figure S8** Avocado oil weathered on day twelve classifying correctly.


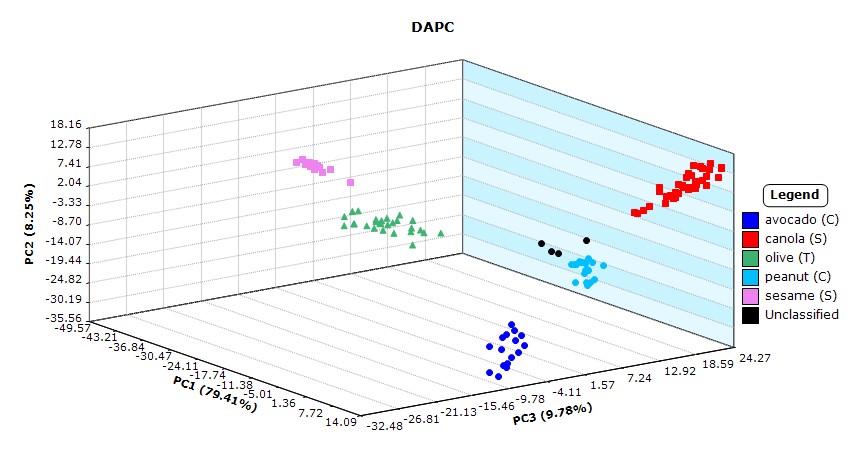

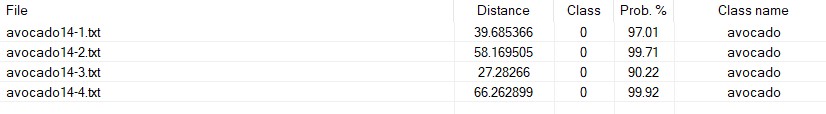


**Figure S9** Avocado oil weathered on day nineteen classifying correctly.


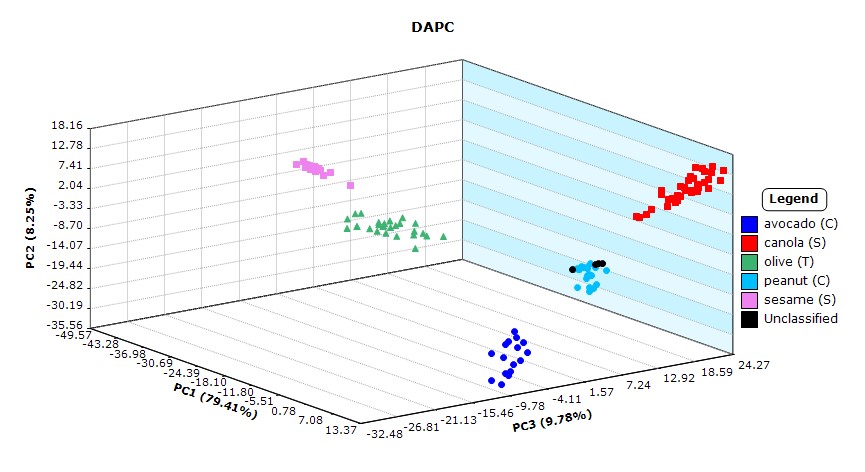

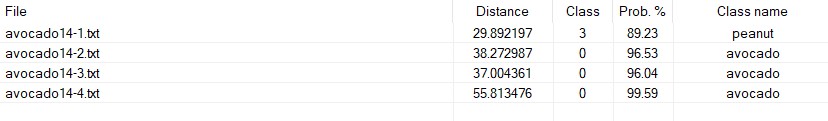
**Figure S10** Avocado oil weathered on day 26 classifying incorrectly.


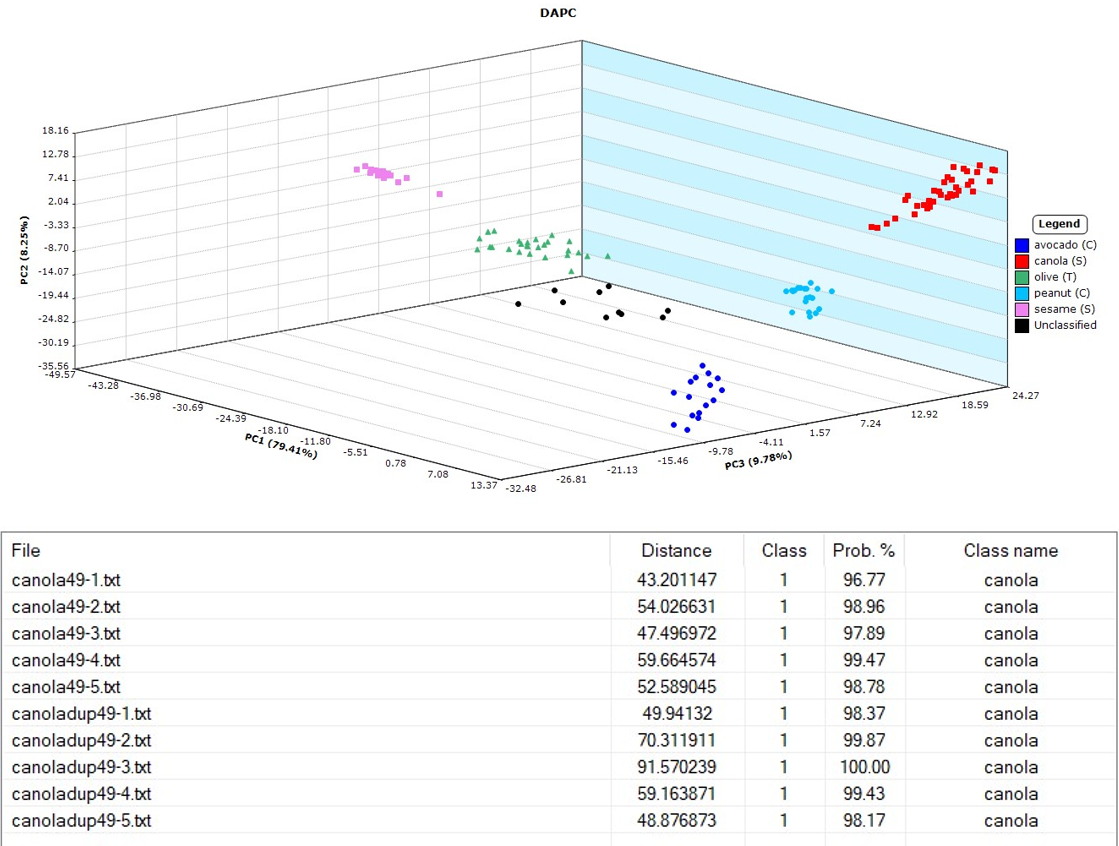


**Figure S11** Canola oil weathered on day 5 classifying correctly.


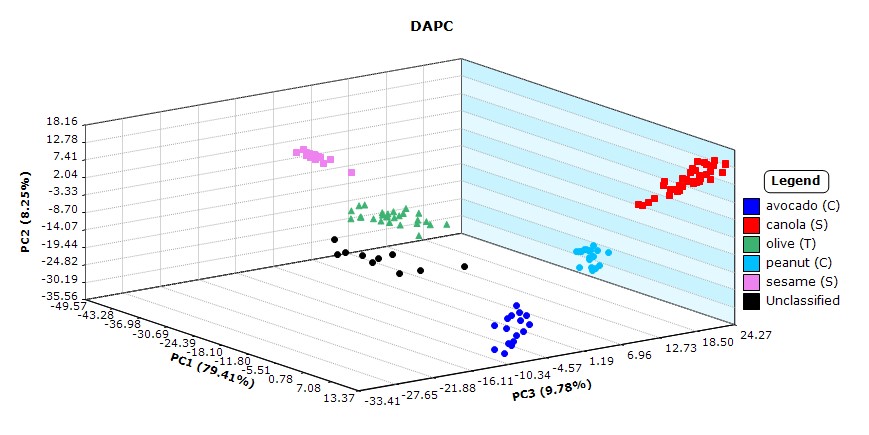

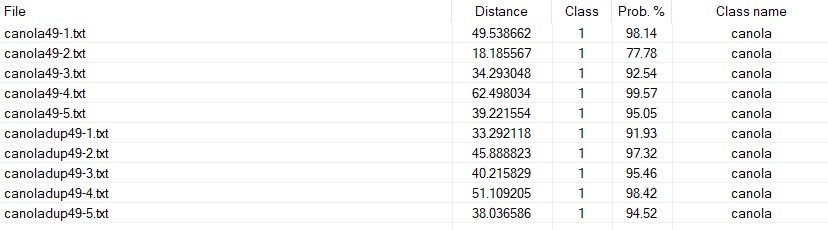


**Figure S12** Canola oil weathered on day 12 classifying correctly.


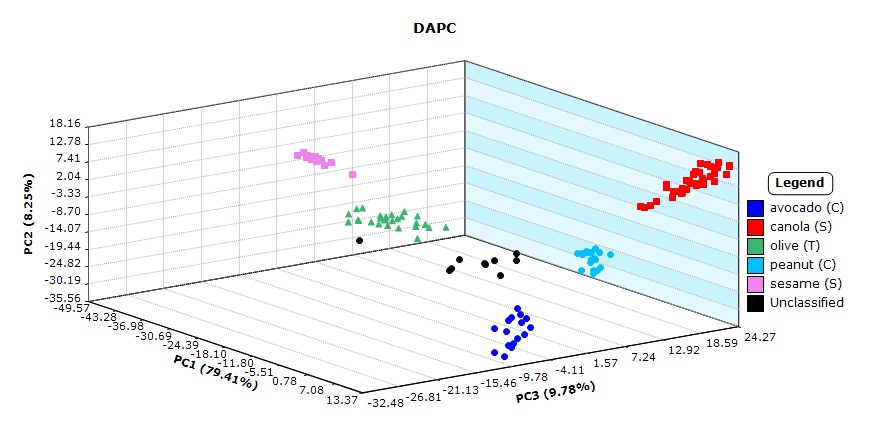

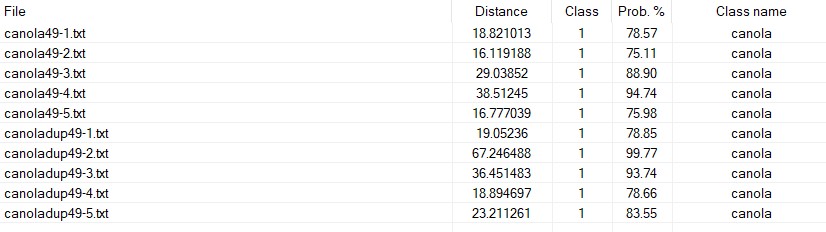


**Figure S13** Canola oil weathered on day 19 classifying correctly.


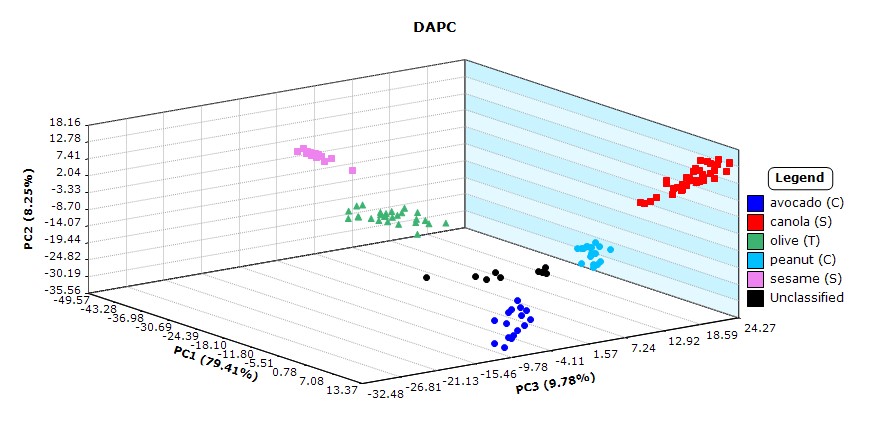

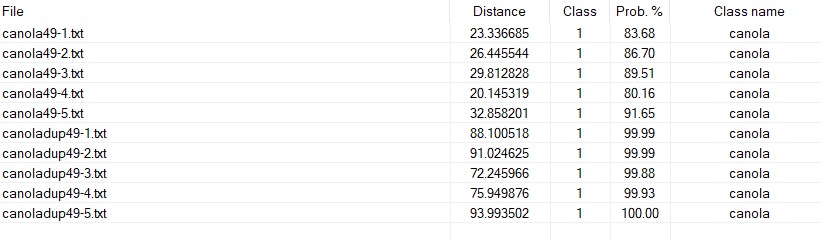


**Figure S14** Canola oil weathered on day 26 classifying correctly.


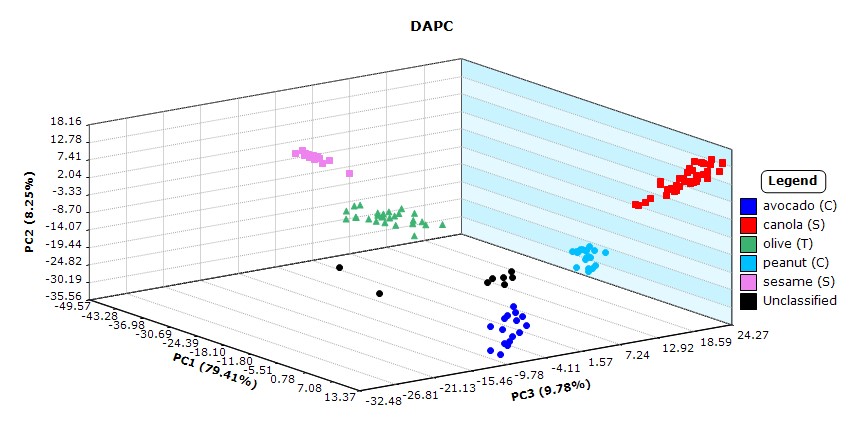

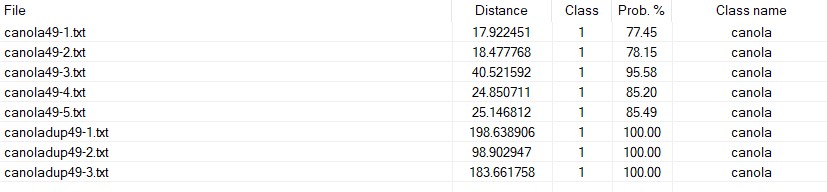


**Figure S15** Canola oil weathered on day 33 classifying correctly.


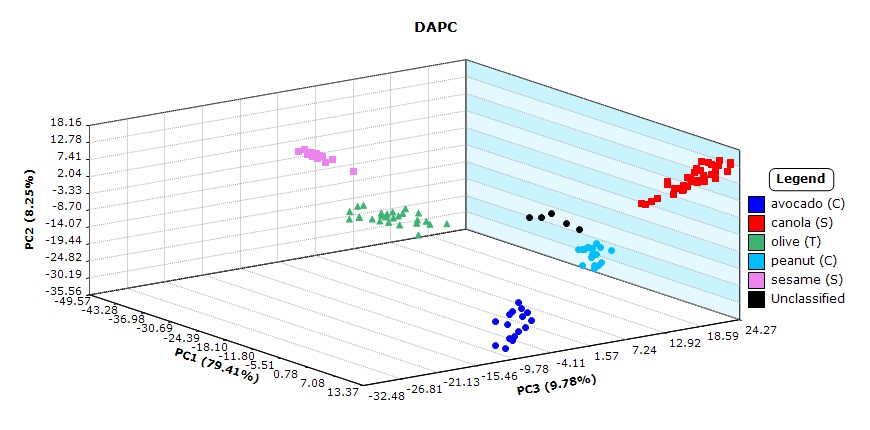

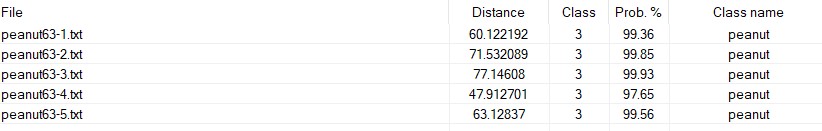


**Figure S16** Peanut oil weathered on day 5 classifying correctly.


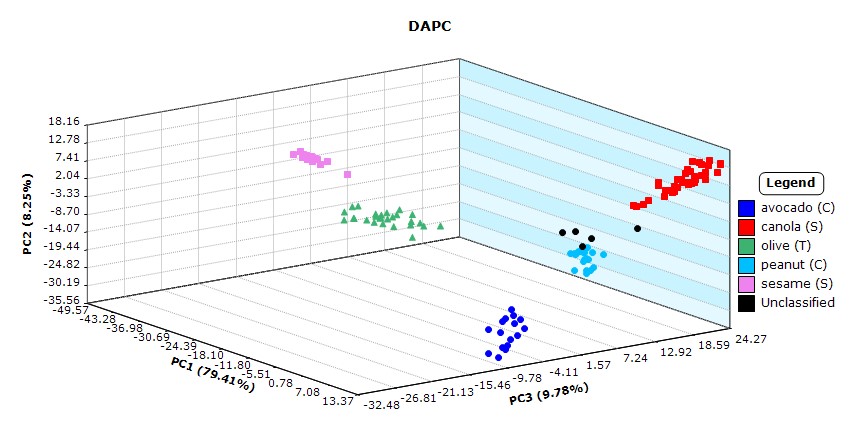

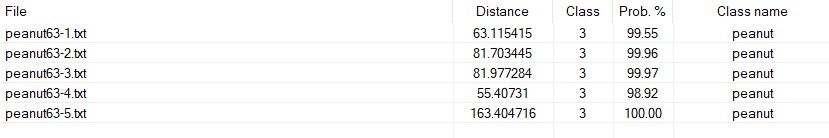


**Figure S17** Peanut oil weathered on day 12 classifying correctly.


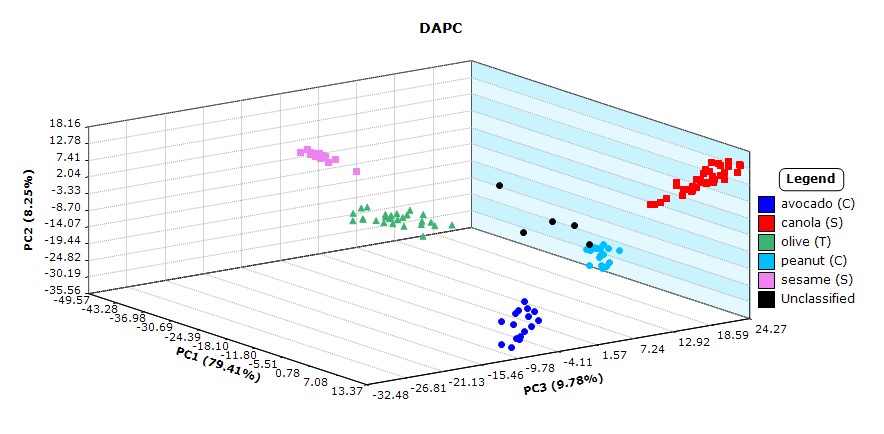

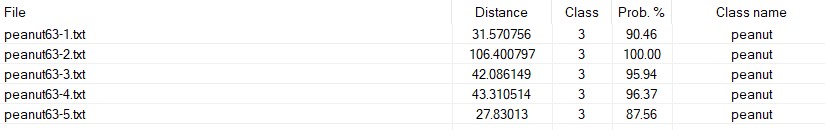


**Figure S18** Peanut oil weathered on day 19 classifying correctly.


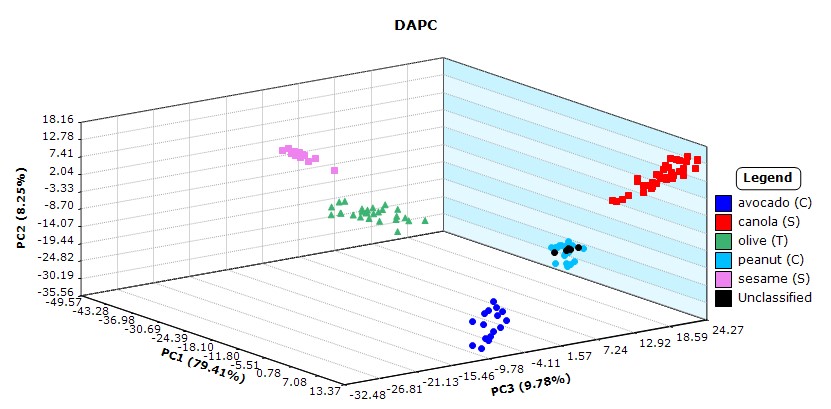

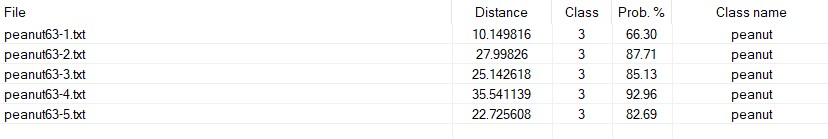


**Figure S19** Peanut oil weathered on day 26 classifying correctly.


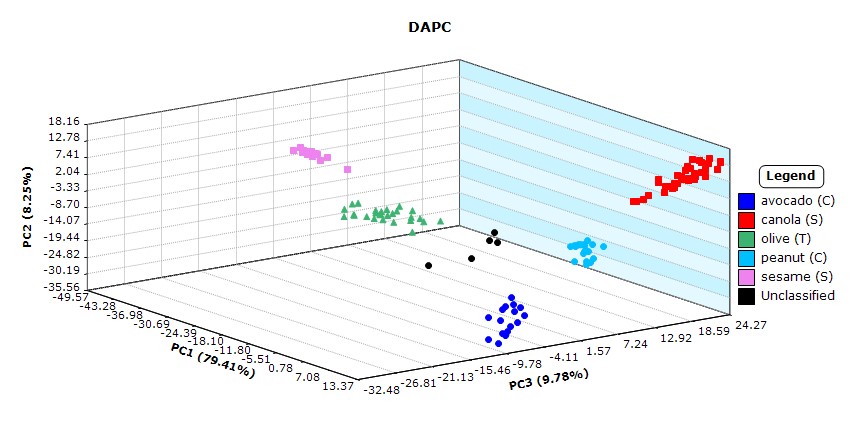

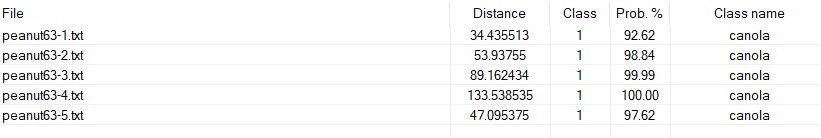


**Figure S20** Peanut oil weathered on day 33 classifying incorrectly.
